# Genome-Wide Methylome Analyses Reveal Novel Epigenetic Regulation Patterns in Schizophrenia and Bipolar Disorder

**DOI:** 10.1155/2015/201587

**Published:** 2015-02-04

**Authors:** Yongsheng Li, Cynthia Camarillo, Juan Xu, Tania Bedard Arana, Yun Xiao, Zheng Zhao, Hong Chen, Mercedes Ramirez, Juan Zavala, Michael A. Escamilla, Regina Armas, Ricardo Mendoza, Alfonso Ontiveros, Humberto Nicolini, Alvaro Antonio Jerez Magaña, Lewis P. Rubin, Xia Li, Chun Xu

**Affiliations:** ^1^College of Bioinformatics Science and Technology, Harbin Medical University, Harbin 150086, China; ^2^Center of Excellence in Neuroscience and Department of Psychiatry, Paul L. Foster School of Medicine, Texas Tech University Health Science Center, El Paso, TX 79905, USA; ^3^Department of Pediatrics, Paul L. Foster School of Medicine, Texas Tech University Health Science Center, El Paso, TX 79905, USA; ^4^Department of Psychiatry, University of California at San Francisco, San Francisco, CA 94103, USA; ^5^Los Angeles Biomedical Research Center at Harbor, University of California Los Angeles Medical Center, Torrance, CA 90502, USA; ^6^Instituto de Informacion de Investigacion en Salud Mental, 64710 Monterrey, NL, Mexico; ^7^Medical and Family Research Group, Carracci S.C., 03740 Mexico City, DF, Mexico; ^8^Centro Internacional de Trastornos Afectivos y de la Conducta Adictiva (CITACA), 01010 Guatemala City, Guatemala

## Abstract

Schizophrenia (SZ) and bipolar disorder (BP) are complex genetic disorders. Their appearance is also likely informed by as yet only partially described epigenetic contributions. Using a sequencing-based method for genome-wide analysis, we quantitatively compared the blood DNA methylation landscapes in SZ and BP subjects to control, both in an understudied population, Hispanics along the US-Mexico border. Remarkably, we identified thousands of differentially methylated regions for SZ and BP preferentially located in promoters 3′-UTRs and 5′-UTRs of genes. Distinct patterns of aberrant methylation of promoter sequences were located surrounding transcription start sites. In these instances, aberrant methylation occurred in CpG islands (CGIs) as well as in flanking regions as well as in CGI sparse promoters. Pathway analysis of genes displaying these distinct aberrant promoter methylation patterns showed enhancement of epigenetic changes in numerous genes previously related to psychiatric disorders and neurodevelopment. Integration of gene expression data further suggests that in SZ aberrant promoter methylation is significantly associated with altered gene transcription. In particular, we found significant associations between (1) promoter CGIs hypermethylation with gene repression and (2) CGI 3′-shore hypomethylation with increased gene expression. Finally, we constructed a specific methylation analysis platform that facilitates viewing and comparing aberrant genome methylation in human neuropsychiatric disorders.

## 1. Introduction

Schizophrenia (SZ) and bipolar disorder (BP) are complex mental diseases. Similar to cancer or diabetes, these neuropsychiatric disorders aggregate in families but do not segregate in a strictly Mendelian manner [1, 2]. Over the past decades, numerous genetic association and linkage studies have shed light on molecular pathways involved in SZ and BP [3–5]. However, limitations of replication and identification of risk alleles having only small effect sizes suggest that nongenetic factors are also important in these disorders [6, 7]. Recently, increasing emphasis is being focused on the potential roles of epigenetic variation in the etiopathogenesis of SZ and BP.

DNA methylation is a key epigenetic mechanism in developmental regulation of gene expression. Evidence indicates that DNA methylation is important in several neurobiological and cognitive processes [8], including neurogenesis and brain development [9], neuronal activity [10], and learning and memory [11]. Prompted by these observations, it is plausible that aberrant DNA methylation, frequently implicated in cancers, also may be a contributor in a spectrum of psychiatric disorders including SZ and BP [12].

Increasingly, investigation of epigenetic variation in psychiatric disorders [13] is uncovering aberrant methylation states in several genes identified from genetic association studies. Disease-associated methylation variants in* Reelin* [14, 15],* Sox10* [16], and* Foxp2* [17] have been identified from postmortem brain and peripheral blood specimens. The advent of genome-wide DNA methylome analysis now facilitates exploring aberrant DNA methylation in psychiatric disorders. Mill et al. performed the first epigenome-wide characterization of DNA methylation in individuals having major psychosis; they surveyed 12,000 GC-rich regions, including CpG islands (CGIs), obtained from prefrontal cortical brain tissue [18]. This study identified several dozen aberrant DNA methylation sites in genes known to be involved in brain development and neurotransmitter signaling.

Investigation of monozygotic twins who are discordant for psychiatric disease is another powerful strategy for uncovering disease-associated epigenetic changes. Petronis et al. found* Drd2* was differently methylated in a twin pair discordant for SZ [19]. In two twin pairs discordant for BP, Kuratomi et al. found increased methylation upstream of the* Sms* gene and decreased methylation upstream of* Ppiel* [20]. At the time of this writing, the most comprehensive twin study using systematic genome-wide analysis of DNA methylation differences employed whole blood DNA microarray-based profiling; several of the aberrantly methylated genes identified have been implicated in psychiatric disorders including SZ and BP [21].

As a consequence of a focus on specific genes of interest and genomic regions of suspected functional relevance, for example, promoters and related CGIs, only a small fraction of human CpGs has been interrogated. To date, unbiased methylome-wide approaches for studying the global genomic distribution of aberrant methylation sites in SZ and BP have been limited [22]. Fortunately, the development of next-generation sequencing now facilitates assessment of genome-wide epigenetic changes without the limitations of probe-based microarray platforms. Methyl-DNA immunoprecipitation in association with high-throughput sequencing (MeDIP-Seq) is a genome-wide mapping strategy that has been successfully used to profile DNA methylation patterns in several human cancers [23, 24].

In this study, we used MeDIP-Seq to investigate the whole-genome distribution of aberrant DNA methylation in six schizophrenia samples and three bipolar disorder samples and compared these with the methylation patterns of a normal sample. We observed distinct patterns of aberrant DNA methylation around transcriptional start sites (TSS) frequently occurring not in CGIs, but rather in sequences up to 2 kb distant from a CGI, termed “CpG island shores” [25], as well as in promoters that lack CGIs. In addition, this study has uncovered several hundred novel SZ- and BP-associated aberrantly methylated genes. These gene functions include long-term potentiation, metabolism, and signaling pathways. The comprehensive psychiatric disorder methylome map here generated specifies precise genomic locations that undergo methylation changes, which should be a valuable resource for understanding epigenetic regulation of the psychosis disease genome.

## 2. Materials and Methods

### 2.1. Clinical Peripheral Blood Samples

All subjects had ancestry from Mexico or Central America and were medication-free. Genomic DNA was extracted from blood as detailed previously [26]. Subjects were designated as affected if they met best estimate consensus diagnoses for either BP or SZ using DSM-IV-TR criteria; the control sample had no history of an Axis I disorder. These studies were approved by the Institutional Review Board of TTUHSC and participating institutions in the United States, Guatemala, and Mexico. Written informed consent was obtained from all participants.

### 2.2. MeDIP-Seq

Genomic DNA was fragmented into 100–500 bp by sonication. DNA ends were repaired to overhang a 3′-dA, and then adapters were ligated to the DNA fragment ends. Double-stranded DNA was denatured and DNA fragments were immunoprecipitated using a 5-mC antibody. Real-time PCR was used to validate immunoprecipitation quality. DNA fragments of proper size (usually 200–300 bp, including adaptor sequence) were selected after PCR amplification. Finally, the resultant libraries were used for sequencing. All raw sequencing data have been submitted to the NCBI SRA database (Accession: SRP046293).

The human genome sequence and mapping information were downloaded from the University of California Santa Cruz Genome Bioinformatics Site (UCSC, http://genome.ucsc.edu/). MeDIP-Seq data were mapped to the reference genome using SOAP2 software [27]. Only unique alignments having no more than 2 mismatches were considered in further analysis.

### 2.3. Genomic Features Annotation

The genomic coordinates for the investigated human genomic features were downloaded from the UCSC public database. RefSeq gene promoters were defined as ±2 kb of sequence flanking transcription start sites, as in previous studies. In addition, in order to investigate the methylation patterns of miRNA promoters, we defined putative miRNA promoters as the 2 kb upstream of miRNA precursors. Table* CpGislandext* (UCSC) was used for the set of CpG islands. We excluded CGIs with “random” chromosome location. Following Irizarry et al., the CpG island shores are defined as the 2 kb regions nearby CGIs [25].

### 2.4. Global DNA Methylation Analysis

The genome was divided into 10 kb segments. The read depth (AM) for each segment was calculated and the read count of each segment was normalized with this formula:

(1) $$\begin{array}{c} \text{AM}=\frac{\text{RC}\times 10^{6}}{\text{URC}}, \end{array}$$

where RC is read count of the distinct 10 kb length segment and URC is the number of unique mapped reads in the sample.

### 2.5. Peak Scanning

Whole genome peak scanning was based on a defined analysis model [28]. Dynamic Poisson distributions were used to calculate the *P* value of a specific region based on the number of unique mapped reads. A region with a *P*  value < 1.0*e* − 5 is defined as a peak.

### 2.6. DMR Identification between Multiple Samples Based on Peak

Peaks of two samples identified as above were merged as candidate differentially methylated regions (DMR). For each candidate DMR, the number of reads for each sample was calculated. Then, numbers of reads were assessed by Chi-square tests to obtain DMRs. For a candidate region, the value of Chi-square is calculated as follows:

(2) $$\begin{array}{c} \chi^{2}=\frac{{\left(\left|a*d-b*c\right|-n/2\right)}^{2}*n}{\left(a+b\right)*\left(c+d\right)*\left(a+c\right)*\left(b+d\right)}, \end{array}$$

where “*a*” and “*c*” are the number of reads mapped to the specific region in the normal and affected samples and “*b*” and “*d*” are the number of reads mapped to other regions; “*n*” is the total number of reads in normal and affected samples. The resultant regions had a FDR less than 5% and the difference of reads numbers was more than twice that considered as DMRs.

To explore whether DMRs were enriched in certain chromosome or structural genomic features, the epitools R-package was used to compute odds ratios for specific genomic features (e.g., promoters) against all other features. The significance of odds ratio values was calculated using Fisher's exact test. The enriched chromosome bands were identified in the same way.

### 2.7. Identification of Aberrant Promoter Methylation Patterns

To investigate the methylation patterns of aberrant methylated gene promoters in SZ and BP, each promoter region was first divided into forty windows. Whenever a window was covered by methylation peaks, we considered the window “methylated” and marked it as “1”; otherwise, the window was regarded as unmethylated and marked as “0.” We focused on two types of gene promoters: (a) those specifically aberrantly methylated in SZ samples and (b) those methylated only in the normal sample. Aberrant methylation patterns were analyzed in the context of CGIs. Finally, five methylation patterns were identified for promoters showing specifically aberrant methylation in at least four of the SZ samples: (1) methylation was mostly confined to CGIs and the number of overlapped windows between “methylated” and “covered by CGIs” was larger than half of the number of methylated windows or half of those covered by CGIs; (2) methylation was positioned 5′ to the CGIs; (3) methylation was positioned 3′ to the CGIs; (4) methylation overlapped with CGIs; and (5) the aberrantly methylated promoters lacked CGIs.

### 2.8. Gene Expression Analysis

RNA-Seq was performed to profile gene expression in the one normal and three schizophrenia samples. Oligo (dT) beads were used to isolate poly(A) mRNA from total RNA from these samples. Fragmentation buffer was added and the resulting 200~300 bp fragments were used as templates for random hexamer-primer synthesis of first-strand cDNAs. Second-strand cDNA was synthesized using buffer, dNTPs, RNase H, and DNA polymerase I. Fragments were purified with a QIAquick PCR extraction kit and resolved with EB buffer for end reparation and adding poly(A). Based on the results of agarose gel electrophoresis, fragments were connected with sequencing adaptors; PCR was performed by selecting suitable fragments as templates. The library was sequenced as paired-end 90 bp reads using IlluminaHiseq 2000. Clean reads were mapped to the reference genome and respective gene sequences using SOAP2. Mismatches of no more than two bases were accepted in the alignments. Reads per kilobase of model per million base pairs sequenced (RPKM) were used to quantify the gene expression level as described in detail by Mortazavi et al. [29]. For genes having more than one transcript, the longest one was used to calculate the expression level.

### 2.9. Gene Set Enrichment Analysis

Gene Set Enrichment Analysis (GSEA) is a computational method that determines if a set of genes defined* a priori* shows statistically significant, concordant differences between two biological states (e.g., phenotypes) [30, 31]. Gene expression in the normal and SZ samples was profiled by RNA-Seq and the data uploaded to GSEA. Enrichment analysis was performed using aberrant methylation target gene lists, such as aberrantly methylated high CpG content promoters (HCPs), intermediate CpGs (ICPs), low CpGs (LCPs), or gene sets having distinct aberrant methylation patterns.

### 2.10. Functional Pathways Identification

KEGG analysis was performed to find enriched pathways using Gene Set Analysis Toolkit V2 (http://bioinfo.vanderbilt.edu/webgestalt/), which is based on hypergeometric tests. *P* values were derived from multiple tests corrected in order to reduce false-positive rates. KEGG pathways having adjusted *P* values of < 0.05 and with at least two interesting genes were considered significant.

## 3. Results

### 3.1. The Landscape of DNA Methylation in SZ and BP

**Figure 1:**
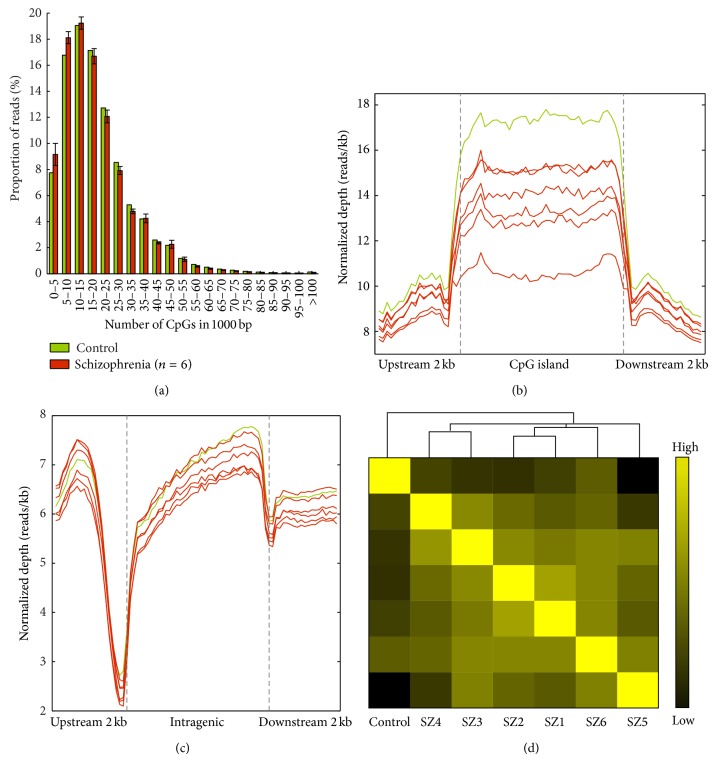
The DNA methylation landscape of schizophrenia. (a) Distribution of genome varies density of CpG. The *x*-axis indicated the range of number of CpGs in 1 kb and the *y*-axis indicates the proportion of reads in each specific range. After dividing the genome into 1 kb windows, we calculated the distribution of DNA methylation windows, which showed various CpG densities. Most reads cluster in regions that have a low number of CpGs. (b) Distribution of reads around CpG islands. The methylation level of CGIs is higher than of CGI shores. The upstream and downstream 2 kb of CGIs were divided into twenty equal regions, and CGIs were divided into forty equal regions, respectively. For each region, the normalized read number was calculated. (c) Distribution of reads around TSSs, which reflects the TSS hypomethylation and gene body methylation. (d) Pairwise similarity of DNA methylation among six schizophrenia subjects and a normal control. Pearson correlation coefficients of genome-wide methylation are displayed according to the color scale.

We performed comprehensive blood DNA methylation profiling of one control, six SZ, and three BP subjects using MeDIP-Seq (Table 1). A total of 73.5 million paired-end reads per sample were generated and 71.8% of reads were uniquely aligned to the human genome. Within the 1 kb genomic DNA windows, we observed different densities of CpGs and found that most reads for the SZ samples clustered in regions containing a low number of CpGs (Figure 1(a)). This result indicates that the MeDIP coverage was not low, even for regions of low CpG density, and that we successfully recovered a considerable fraction of methylated regions. In addition, we found that the distributions of methylation levels around the CGIs and TSSs were similar to that found in previous studies [32] (Figures 1(b) and 1(c)). These internal validations collectively support the sequencing strategy and results.

To obtain an overview of the methylation maps and to explore correlations of methylation among samples, we divided the entire genome into 10 kb segments and counted the number of reads mapped to each segment. To compare the methylation levels among samples, the read count of each segment was normalized against the total number of reads in corresponding samples. Pairwise comparison of genome-wide methylation across all samples showed a high correlation among all six SZ samples (*R* > 0.972), but not with the control, suggesting broadly altered methylation levels in SZ (Figure 1(d)). We observed similar findings for BP (Supplementary Figure  1 in Supplementary Material available online at http://dx.doi.org/10.1155/2015/201587).

### 3.2. Genomic Distribution of Aberrant DNA Methylation in SZ and BP

**Figure 2:**
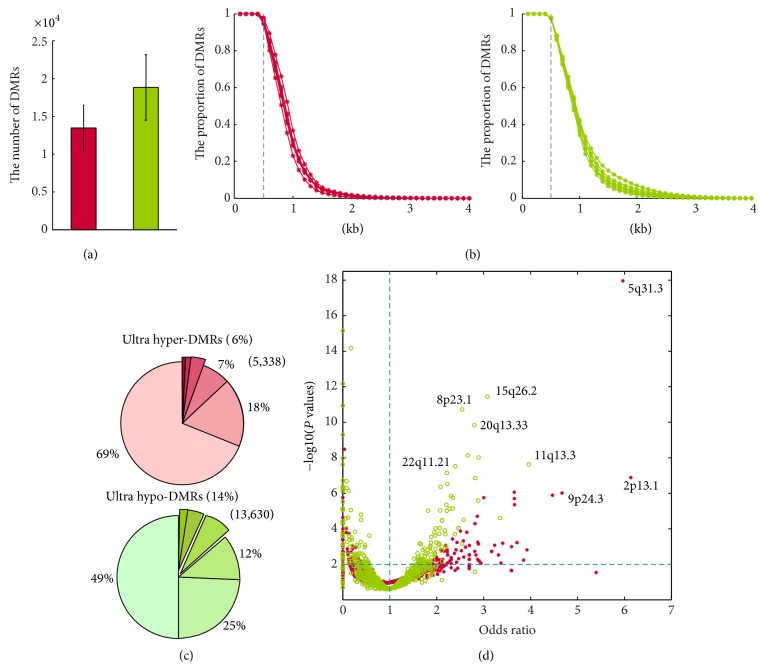
Genomic distribution of DMRs in schizophrenia (SZ). (a) The number of differentially methylated regions was counted for each SZ sample. The* red bar* indicates hypermethylated DMRs and the* green bar* indicates hypomethylated DMRs. (b) The cumulative distribution of lengths of DMRs for hyper- or hypomethylated DMRs.* Red lines* indicate hypermethylated and* green lines* hypomethylated DMRs. (c) Pie chart representing the proportions of DMRs in SZ samples. The darker the color shade, the greater the number of samples. A segment is regarded as differentially methylated if the overlap length was larger than 250 bp. (d) Chromosome band enrichment of ultra DMRs.* Red stars* indicate hypermethylated chromosome bands and* green circles* indicate hypomethylated bands. The bands indicated in the upper right panel show FDR less than 0.01 and odds ratios greater than one.

Despite global similarities, specific methylation changes were evident in SZ and BP samples. In order to identify the nature of the differences among SZ, BP, and control and to identify DMRs, we carried out pairwise comparisons of six SZ and three BP methylomes with one control methylome. A total of 32,282 DMRs for SZ and 34,933 for BP with a FDR of 5% were identified. They were further subdivided into 13,463 hypermethylated and 18,819 hypomethylated DMRs for SZ (Figure 2(a)) and 10,898 hypermethylated and 24,036 hypomethylated DMRs for BP (Supplementary Figure  2A). To exclude individual variations and to identify “ultra DMRs” (defined as genomic regions that are hyper- or hypomethylated in more than half of samples), we first explored the distribution lengths of all DMRs. Interestingly, nearly 95% of all DMRs were larger than 500 bp (Figure 2(b) and Supplementary Figure  2B). Therefore, we divided the genome into 500 bp segments to identify the ultra DMRs. A total of 5,338 (6%) and 9,291 (21%) ultra hypermethylated DMRs were identified in SZ and BP, respectively; 13,630 (14%) for SZ and 28,410 (36%) for BP ultra-hypomethylated DMRs were identified and explored further (Figure 2(c) and Supplementary Figure  2C). In agreement with reports for several cancers [24, 33], we observed that the number of hypomethylated DMRs is greater than that of hypermethylated DMRs, indicating a global hypomethylation and local hypermethylation in psychiatric disorders.

We next examined the genomic distributions of these ultra DMRs. Hypomethylated and hypermethylated regions were distributed throughout the entire genome. However, 9.49% and 11.10% chromosome loci were enriched with hyper- and/or hypomethylated DMRs. In particular, some ultra DMRs were clustered at specific loci, including chromosomal loci for SZ (Figure 2(d)) and BP (Supplementary Figure  2D) and loci shared between the disorders (20q13.33 and 5p31.3). These ultra DMR regions span several known SZ- and BP-associated genes including* Smarca2* [34] and* Comt* [35]. The* Smarca2* gene, located on chromosome 9, is a member of the SWI/SNF complex and has been implicated in regulation of gene expression, cell cycle control, and oncogenesis. An association between SZ and three SNPs in two linkage disequilibrium blocks of the* Smarca2* gene has been reported in a Japanese population [36]. In addition, SZ risk alleles have been associated with low* Smarca2* expression levels in postmortem prefrontal cortex. The current analysis identified two hypermethylated DMRs near* Smarca2*, providing additional evidence for its epigenetic regulation in SZ. The* Comt* gene, located on 22q11.2, is involved in inactivation of catecholamine neurotransmitters (dopamine, epinephrine, and norepinephrine). It has been proposed that an inherited variant of* Comt* carries a predisposition to schizophrenia in later life [37].

### 3.3. Target Positioning of Aberrant Methylation in SZ and BP

The discovery of aberrant DNA methylation in complex diseases, especially in cancer, has focused investigation on specific genes of interest and on genomic regions assumed to be important functionally, such as promoters and CpG islands [38, 39]. In order to provide a more systematic landscape of methylation in SZ, we mapped all DMRs to their nearest genomic features and performed an enrichment analysis on those genomic elements that are associated with DMRs. Analysis of the methylation level of these genomic features showed the promoters and CGIs having increased methylation. Consistent with previous studies in cancer, we also observed global hypomethylation in repeat elements, such as SINE, LINE, and LTR (Supplementary Figure  3 and Supplementary Figure  4A). Although the majority of differentially methylated DMRs occur outside CGIs, we also identified promoters, CGI, and CGI shores significantly associated with hyper- and hypomethylated DMRs (adjusted *P* < 0.01), emphasizing roles of DNA methylation in these genomic features. Although the observed numbers of repeat elements did not statistically differ, functional relevance cannot be excluded (Supplementary Figure  5 and Supplementary Figure  4B).

**Figure 3:**
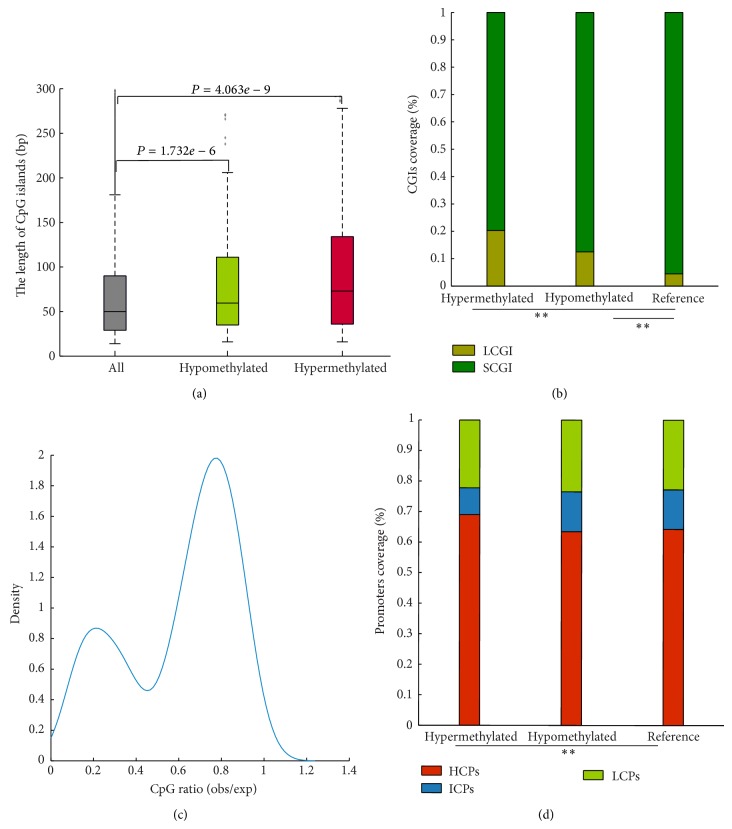
Ultra DMRs are associated with long CGIs and HCPs. (a) CGI length distribution for all CGIs, hypermethylated CGIs, and hypomethylated CGIs. (b) Hyper- or hypomethylated ultra DMRs are enriched in LCGI promoters (*P* values < 0.01, Fisher exact test). (c) The distribution of CpG ratios for aberrantly methylated promoters. (d) Hypermethylated ultra DMRs are enriched in HCPs.

CGIs in the human genome vary ~30-fold in length. Lengths of CGIs have functional consequences; genes containing long CGI genes are preferentially associated with developmental and regulatory functions [40]. We found that CGIs associated with DMRs were significantly overrepresented in long CGIs, indicating potentially important roles in SZ (Figures 3(a) and 3(b)) and BP (Supplementary Figures  6A and  6B) and confirming previous observations in SZ [41]. Despite a modest bias of aberrant methylation toward long CGIs, the distribution of promoters' CpG content is bimodal, revealing two distinct populations having high or low CpG frequency (Figure 3(c) and Supplementary Figure  6C).

To explore further relationships between methylation and promoter CpG frequency, promoters were divided into three groups based on their CpG ratios [42], that is, LCPs, ICPs, and HCPs. We determined that hypermethylated promoters exhibit an increased proportion of HCPs (Figure 3(d) and Supplementary Figure  6D). The correlation between gene activity and DNA methylation suggests that promoter activity frequency varies among promoter classes, dependent on CpG content. Consequently, HCPs and ICPs are more prone to differential regulation by DNA methylation than are LCPs [42]. Furthermore, genes expressed in most tissues have been reported to be biased toward HCPs [43], suggesting a key role of HCPs in maintenance of basic cellular functions. These results suggest, in SZ and BP, that cellular systems might be regulated via selectively aberrantly methylated genes associated with long CGIs or with high CpG content.

On genomic methylation scanning, we observed a modest number of genes with aberrant promoter methylation occurring in both disorders (Supplementary Figure  7A), including* MPO* on 17q23.1 associated with SZ [44] and SZ/BP [45].* RIMS1* [46] and* SLC30A8* [47] have been associated with the antipsychotic response. These gene products directly interact in a protein interaction network (Supplementary Figure  7B), suggesting functional associations. Our topological analysis indicates that the aberrantly methylated genes in SZ/BP tend to occur at hubs and bottlenecks in protein networks (Supplementary Figures  7C–7E). These results suggest selective methylation of hub and bottleneck genes may be a regulatory mechanism in complex diseases.

### 3.4. Distinct Patterns of Promoter Aberrant Methylation in SZ and BP

Promoter aberrant methylation is proposed to contribute to tumorigenesis via repressing tumor-suppressor gene transcription. Therefore, profiling genome-wide promoter methylation would be expected to identify different patterns of DNA methylation. Our analyses show that promoter regions are enriched in both hyper- and hypomethylated DMRs and imply that a predominance of promoter-centric aberrant epigenetic regulatory effects occurs in SZ and BP.

**Figure 4:**
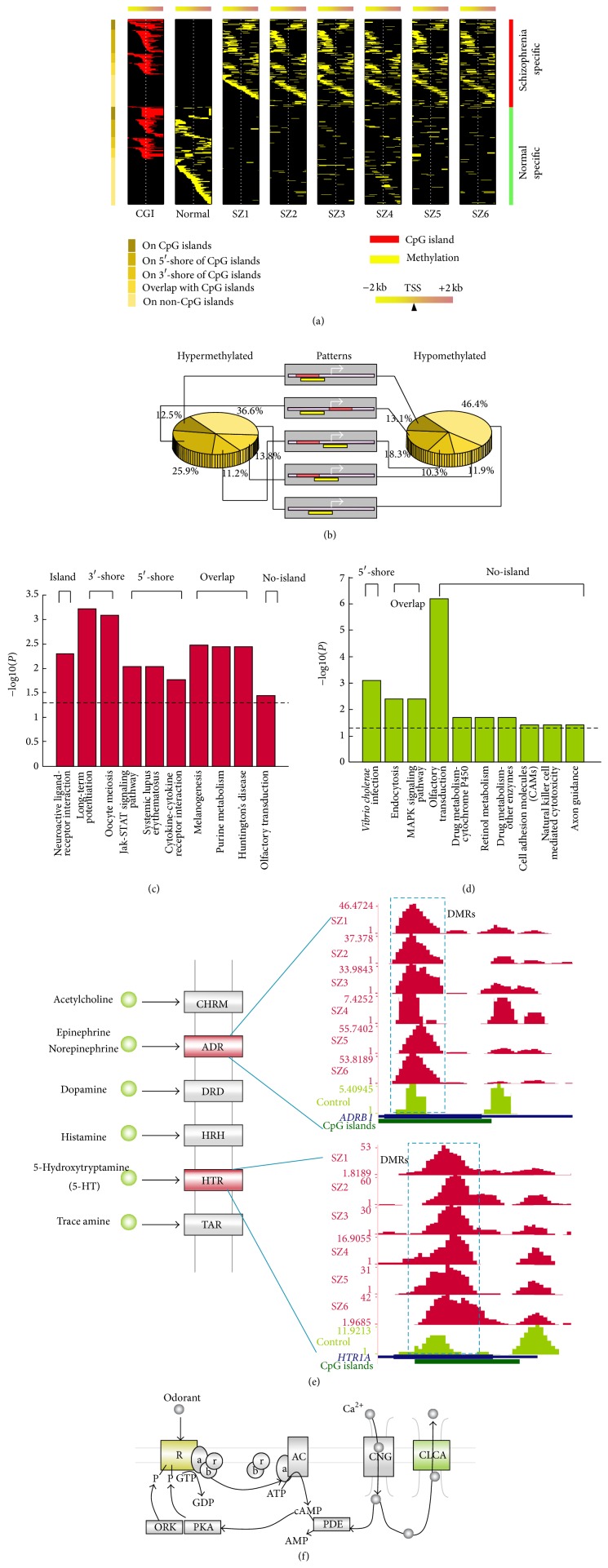
Distinct DNA methylation patterns of promoters in schizophrenia (SZ). (a) Heat map of distinct patterns of promoter methylation. Each row represents a unique promoter region of 100 bp window size, covering ±2000 bp flanking the transcription start site, as indicated by the* white dotted line*. The location of a CGI (*red*) is shown in the first column. Promoters in the top panel are methylated in SZ while those in the lower panel are methylated in normal. Promoters are ordered by the location of methylation as represented with different* shades of brown* on the left. (b) Proportion of distinct patterns of promoter methylation. The middle panel shows a schematic diagram for distinct methylation patterns, while the left and right diagrams indicate the ratios of distinct methylation patterns for hyper- or hypomethylated promoters. (c) KEGG pathways are enriched by hypermethylated genes in SZ. The pathways are enriched by genes with distinct methylation patterns. (d) The KEGG pathways enriched in hypomethylated genes in SZ. (e) Neuroactive ligand-receptor interaction pathways enriched by two hypermethylated genes,* Adrb1* and* Htr1a*. The subfigures show the methylation of these two genes in six SZ samples and one control. (f) The olfactory transduction pathway is aberrantly regulated in SZ; 3 hypermethylated genes are enriched in the receptor while 9 hypomethylated genes at the start and the end of this pathway are enriched.

Next, we focused on the 955 gene promoters that are covered by DMRs in SZ and identified 476 promoters methylated only in SZ or normal control (Supplementary Tables  1 and  2). Visualization of these methylation marks in the context of CpG islands revealed the presence of several distinct methylation patterns on gene promoters. Broadly, promoters fell into two groups based on the presence or absence of a CpG island. Although 41.8% of aberrantly methylated promoters lacked CpG islands, they exhibited aberrant methylation around the TSS. The remaining 58.2% of aberrantly methylated promoters had CGIs spanning the TSS and showed four other distinct methylation patterns (Figure 4(a)): (1) methylation was mostly confined to the CGIs; (2) methylation was positioned 5′ to the CGIs; (3) methylation was positioned 3′ to the CGIs; (4) methylation overlapped with CGIs. Despite the observation that, in some promoters, aberrant methylation was confined to the CGIs, the genome-wide analyses of the promoter methylation patterns enabled us to discover an unexpected physical relationship between CGIs and aberrant DNA methylation in SZ; namely, 87.5% of the hypermethylated and 86.9% of the hypomethylated DMRs were located at the shore of CGIs in promoters or in those promoters lacking CGIs (Figure 4(b)). Although we determined 129/199 gene promoters overlapped with hyper- or hypomethylated DMRs (Supplementary Table  3) in BP, few of these gene promoters were specifically methylated in BP. Aberrant methylated promoter patterns will be much clearer with more BP samples in a future study.

The identification of these aberrantly methylated regions suggests the need for further functional studies, such as the mechanisms of how aberrant DNA methylation is targeted to these regions and the role of aberrant methylation in CGI shores.

### 3.5. Aberrantly Methylated Genes Are Enriched for Pathways Relevant to SZ and BP

In order to determine potential functional significance of the distinct DNA methylation patterning observed above, we performed pathway enrichment analyses (*P*_adjusted_ < 0.05). The results indicate hypermethylated CGI genes are enriched for neuroactive ligand-receptor interactions and genes aberrantly methylated in the shore of CGIs are preferentially involved in long-term potentiation and the Jak-STAT signaling pathway in SZ (Figure 4(c), Supplementary Table  4). In contrast, hypomethylation in CGI shores showed enrichment for genes involved in infection, while genes lacking CGIs were enriched in metabolism, cell adhesion, and axon guidance (Figure 4(d), Supplementary Table  5). These results suggest that genes with distinct aberrant methylation patterns might affect several pathophysiological pathways in SZ.

Neuroactive ligand-receptor interactions [48] and long-term potentiation [49] are directly associated with neurodevelopment and SZ. The long-term potentiation pathway is critical in synaptic plasticity and is associated with SZ. Interestingly, we found aberrantly hypermethylated genes clustered in the Rhodesian-like amine GPCR family (Figure 4(e)). In particular,* ADRB1* and* HTR1a* were hypermethylated in 5/6 SZ samples. Associations between* HTR1a* and SZ, implicating a role in SZ pathophysiology, have been reported [50]. Moreover, we found that the olfactory transduction pathway is enriched in hyper- and hypomethylated genes lacking CGIs and is enriched at several locations of this pathway in SZ (Figure 4(f)), again indicating potential for multiple aberrations in this pathway that may contribute to SZ.

Although no genes with specific methylated patterns were identified in BP, the genes that overlapped with DMRs highlighted pathways associated with cell signaling and metabolism (Supplementary Table  5). These results suggest that aberrant methylation may have an impact on SZ and/or BP pathophysiology, mainly by targeting key nodes of involved pathways.

### 3.6. DNA Methylation Code and Transcriptional Regulation in SZ

**Figure 5:**
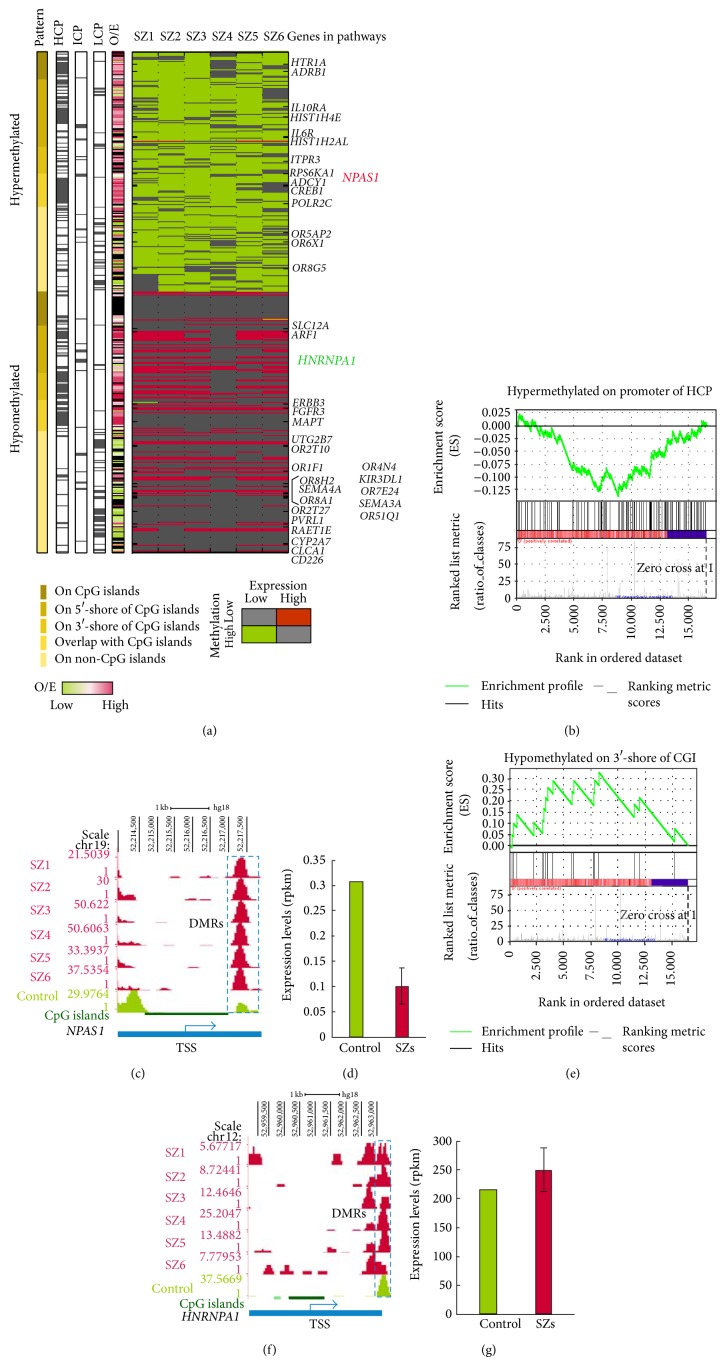
DNA methylation code and gene expression. (a) The global view of DNA methylation code and gene expression. The heat map is based on levels of gene expression and DNA methylation at each gene locus; a key to the color coding is presented below the map. The table on the left designates the pattern of aberrant promoter methylation and the CpG ratios. (b) HCP methylation is associated with gene repression. Gene Set Enrichment Analysis (GSEA) of HCPs hypermethylated in SZ was performed on expression data from RNA-Seq. The correlation between gene expression and promoter methylation is *P* < 0.045. (c) DNA methylation in the* Npas1* gene promoter in 6 SZ and normal samples. Promoter regions are marked with* pink bars* and CGIs with* green*. The* blue rectangle* with dashed lines indicates the DMR regions:* red*, SZ samples;* green*, normal control. (d)* Npas1* gene expression in normal (*blue*) and SZ (*dark red*) samples. (e) Hypomethylation of promoter 3′-shores of CGIs is associated with increased gene expression (*P* value < 0.040). (f) DNA methylation in the* Hnrnpa1* gene promoter. Promoter regions are marked with* pink bars *and CGIs with* green*. The* blue rectangle* with dashed lines indicates the DMR regions:* dark red*, SZ samples;* blue*, normal control. (g)* Hnrnpa1* gene expression in normal (*blue*) and SZ (*dark red*) samples.

The relationship between promoter methylation and transcriptional repression of downstream genes has been established in some human diseases [51, 52], but not previously in SZ/BP. Consequently, we next inquired if promoter methylation events are associated with transcriptional changes. We found the relationship between DNA methylation and gene expression in most hypermethylated genes in this project showed lower levels of expression in SZ (Figure 5(a)). By analyzing genes with distinct CpG content or with distinct methylation patterns, we found that promoters with high CpG ratios and that were hypermethylated in SZ were significantly associated with gene repression (*P* < 0.045) using GSEA (Figure 5(b)) [30]. Several previously well-characterized SZ-associated genes are also present in our list (Supplementary Table  6). For example,* Npas1*, having a CpG ratio of 0.64, was hypermethylated in 5/6 SZ samples (Figure 5(c)) and its gene expression in these samples was threefold lower than in the control (Figure 5(d)). Transcription factors regulating* Npas3* and* Npas1* gene transcription govern regulatory pathways relevant in SZ [53]. In contrast, genes hypomethylated on a 3′-shore of a promoter CGI tended to be overexpressed (*P* < 0.040, Figure 5(e)), including the* Hnrnpa1* gene (Figure 5(f)), which is highly expressed in the SZ samples (Figure 5(g)).* Hnrnpa1* is a posttranscriptional regulator of gene expression and represses alternative splicing when associated with silencing elements near splice sites. It is implicated in processing primary* Mecp2* RNAs and it binds to telomeric DNA, where it may promote telomere elongation [54]. Thus, aberrant expression of this gene may cause extensive functional abnormalities, and it may be a possible target in SZ diagnosis or treatment.

### 3.7. PDMeth: A Specific Methylation Platform for Human Psychiatric Disorders

We have developed PDMeth, a specific methylation platform for human psychiatric disorders (http://bioinfo.hrbmu.edu.cn/pdmeth). Its focus is the efficient storage and statistical analysis of DNA methylation data specifically related to psychiatric disorders. PDMeth provides integrated gene methylation data based on cross dataset analysis for disease and normal samples and includes a user friendly and configurable genome browser in which multiple genomic and epigenomic resources can be visualized simultaneously. In addition, users can upload their own datasets for comparison with the current SZ/BP samples. In the future, we will continue to extend the database with new methylation datasets.

## 4. Discussion and Conclusion

Although several studies have described specific DNA methylomic changes in psychiatric disorders, knowledge of how changes in DNA methylation impact SZ and BP remains largely limited to effects at several genomic loci. In this study, we provide a comprehensive map of DNA methylation and characterize global genome-wide methylation patterns in SZ and BP. These results support the assertion that epigenetic dysregulation plays an important role in both SZ and BP and provide new important insights into the biological implications of DNA methylation.

First, comparative methylome analysis uncovers numerous potentially important DMRs and methylation patterns, in both intragenic and intergenic regions, associated with SZ and BP. Despite comparable total number of genomic regions methylated in all samples, thousands of specific hypermethylated and hypomethylated DMRs are identified. Consistent with prior studies, we found gene-related genomic features are the predominant targets of aberrant methylation in SZ, such as promoters 5′- and 3′-UTRs [19, 25]. An important observation is that aberrant methylated genes are enriched in promoters having high CpG ratios. Several of these genes have been previously demonstrated to be involved in development and cell regulation. In addition to these gene-related features, the other major target for aberrant methylation is CGIs and CGI shores. This finding extends involvement of CGI shores from cancer [13] to neuropsychiatric disorders.

Further visualization of gene promoter aberrant methylation in the context of CGIs revealed the presence of several distinct methylation patterns in SZ. We found patterns of aberrant promoter methylation that span CGIs, but most methylation events are positioned in the 5′ and 3′ regions flanking CGIs. Emerging evidence suggests these “CGI shores” may play a more important role in the regulation of gene expression than do CGIs themselves. In support of the functional role for CGI shores, GSEA analysis revealed that the aberrant DNA methylation of CGI shores was more strongly associated with gene expression than was aberrant methylation of CGIs. Although the expression data profiled with RNA-Seq correlates well with our methylation data, the conclusions drawn from these integration analyses must be interpreted cautiously since the two data sets were derived from different individuals. We also note that aberrant methylation additionally occurred on promoters that lack CGIs. Previous studies have determined that some genes can be repressed by promoter methylation, despite absence of a CGI in the promoter region. In sum, the promoter aberrant methylation patterns identified here likely regulate the transcriptome in SZ.

Most importantly, we found that 23.8% of genes identified in our study have been previously recognized as associated with SZ. DNA hypo- or hypermethylation changes in 56 genes obtained from peripheral blood samples in our current study are consistent with our recent findings [55] obtained from postmortem brain samples from patients with SZ and BP, including* DNMT1, CACNA1S, PRAME, MYT1L*, and* STAB1*. Among these genes,* CACNA1S* on 1q32 [56] and* PRAME* on 22q11.22 [57] are considered “hotspots” for SZ and BP. Moreover, the findings of three SZ-associated genes in our current study, including aberrant hypermethylated* SMAD3*, hypomethylated* ARHGAP26*, and hypermethylated* CREB*, have been confirmed in a recent study that used methyl-CpG-binding domain protein-enriched genome sequencing of the methylated genomic fraction, followed by next-generation DNA sequencing in 759 SZ and 738 controls [58]. The results from our study demonstrate that specific features of methylation profiles in patients with SZ and BP capture signatures of environmental insults in peripheral tissues and, as such, are an important step toward developing diagnostic and therapeutic biomarkers for SZ, BP, and/or other neuropsychiatric conditions [59].

Our findings also indicate that age- and/or sex-associated DNA methylation differences occur within the SZ group. Taking these factors into account and to exclude individual variations, we focused on the ultra DMRs, which were defined as genomic regions that are hyper- or hypomethylated in more than half of samples. In addition, we found 95.6% of hyper-DMRs and 89.9% of hypo-DMRs were identified by comparison with samples similar in age and of the same sex as the control. These results support a conclusion that the majority of our identified DMRs are associated with SZ. An important caveat is our reference to a single control subject. Future investigations will extend the findings in more case and control subjects.

In summary, we have used a high-throughput MeDIP-Seq strategy to characterize the DNA methylome map of schizophrenia and bipolar disease. We observed distinct patterns of DNA methylation around TSSs and have uncovered several hundred novel aberrantly methylated genes in SZ and BP. By incorporating gene expression datasets, we also provide additional evidence that aberrant DNA methylation disturbs gene expression and affects biological pathways in SZ, including neuroactive ligand-receptor interactions and long-term potentiation. The comprehensive psychiatric disorders' methylome map generated here provides precise genomic locations that undergo methylation changes. It should prove to be a valuable public resource for investigations aimed at understanding epigenetic regulation of the SZ and BP genome.

## Supplementary Material

Supplemental Figure S1: The DNA methylation landscape of bipolar disease.
Supplemental Figure S2: Genomic distribution of DMRs in bipolar disease.
Supplemental Figure S3: Ultra DMRs are associated with gene- and CGI-related regions.
Supplemental Figure S4: Ultra DMRs are associated with gene- and CGI-related regions in BP.
Supplemental Figure S5: Ultra DMRs are associated with gene- and CGI-related regions in SZ.
Supplemental Figure S6: Ultra DMRs are associated with long CGIs and HCPs.
Supplemental Figure S7: Common genes aberrant methylated in SZs and BPs.
Supplementary Table 1:The hypermethylated and hypomethylated promoters in SZs.
Supplementary Table 2:The Specific hypermethylated and hypomethylated promoters with distinct patterns in SZs.
Supplementary Table 3: The hypermethylated and hypomethylated promoters in BPs.
Supplementary Table 4: KEGG pathways enriched by aberrant methylated genes specifically in SZs/normal.
Supplementary Table 5: KEGG pathways enriched by aberrant methylated genes in BPs.
Supplementary Table 6: Aberrant hypermethylated HCP genes in SZs.

## Figures and Tables

**Table 1:** The characteristics of normal and schizophrenia patients used in the study.

Type	Sample ID	Age	Sex	Mapped reads	Mapping rate (%)	Unique mapped reads	Unique mapping rate (%)
Control	Control	31	F	63,705,306	86.71	53,558,678	72.90

SZ	SZ1	19	M	65,222,408	88.77	52,602,686	71.60
	SZ2	19	M	64,775,113	88.17	51,767,805	70.46
	SZ3	32	F	64,938,918	88.39	54,367,331	74.00
	SZ4	32	F	64,702,407	88.07	54,361,897	73.99
	SZ5	—	F	64,963,538	88.42	53,367,930	72.64
	SZ6	20	F	64,881,310	88.31	53,296,535	72.54

BP	BP1	39	F	63,027,119	85.79	51,944,268	70.70
	BP2	55	M	64,279,707	87.49	51,104,038	69.56
	BP3	—	M	64,499,638	87.79	51,049,294	69.48

Note: samples SZ1 and SZ2 are monozygous twins; samples SZ3 and SZ4 are monozygous twins.
