# Supplementary material for: Genome-Wide Methylome Analyses Reveal Novel Epigenetic Regulation Patterns in Schizophrenia and Bipolar Disorder
Source: Biomed Res Int. 2015 Feb 4;2015:201587. doi: 10.1155/2015/201587 (PMC4334857; doi:10.1155/2015/201587)
Supplement: Supplementary file 1 — Supplemental Figure S1: The DNA methylation landscape of bipolar disease. Supplemental Figure S2: Genomic distribution of DMRs in bipolar disease. Supplemental Figure S3: Ultra DMRs are associated with gene- and CGI-related regions. Supplemental Figure S4: Ultra DMRs are associated with gene- and CGI-related regions in BP. Supplemental Figure S5: Ultra DMRs are associated with gene- and CGI-related regions in SZ. Supplemental Figure S6: Ultra DMRs are associated with long CGIs and HCPs. Supplemental Figure S7: Common genes aberrant methylated in SZs and BPs. Supplementary Table 1:The hypermethylated and hypomethylated promoters in SZs. Supplementary Table 2:The Specific hypermethylated and hypomethylated promoters with distinct patterns in SZs. Supplementary Table 3: The hypermethylated and hypomethylated promoters in BPs. Supplementary Table 4: KEGG pathways enriched by aberrant methylated genes specifically in SZs/normal. Supplementary Table 5: KEGG pathways enriched by aberrant methylated genes in BPs. Supplementary Table 6: Aberrant hypermethylated HCP genes in SZs. [file 201587.f1.zip › Supplementary files.doc]

**SUPPLEMENTARY INFORMATION**

**Contents: the file contains Supplementary Figures 1 to 7, Supplementary Tables 1 to 6 and Supplementary text.**

**Supplemental Figure S1. The DNA methylation landscape of bipolar disease. (A),** Distribution of genome varies density of CpG. The x axis indicated the range of number of CpG in 1kb and the y axis indicated the proportion of reads in specific range. (**B),** Distribution of reads around CpG Island. (**C),** Distribution of reads around gene body. This figure reflects the methylation level around gene body. (**D),** Pair-wise similarity of DNA methylation among bipolar disease and normal samples. Pearson correlation coefficient of genome-wide methylation is displayed according to the color scale.

**Supplemental Figure S2. Genomic distribution of DMRs in bipolar disease. (A),**The number of differentially methylated regions were counted for each patient with bipolar disorder. Red bar indicates hypermethylated DMRs and green bar indicates hypomethylated DMRs. (**B),** The cumulative distribution of lengths of DMRs for hyper- or hypomethylated DMRs. Red lines for hypermethylated and green lines for hypomethylated DMRs. (**C),** Pie chart representing the proportions of DMRs presented in different number of bipolar disorder samples. The darker color is, the higher number of samples is. (**D),** Chromosome band enrichment of ultra DMRs. Red stars indicate hypermethylated chromosome bands, while green circles indicate hypomethylated bands. The bands in the right up panel are with FDR less than 0.01 and odds ratios larger than one.

**Supplemental Figure S3. Ultra DMRs are associated with gene- and CGI-related regions.** **(A)**, Bar chart representing the number of each genomic feature with methylation signals. Red bar shows the number of features covered by hypermethylated DMRs while green bars for hypomethylated DMRs.

**Supplemental Figure S4.** **Ultra DMRs are associated with gene- and CGI-related regions in BP. (A),** Bar chart representing the number of each genomic feature with methylation signals. Red bar shows the number of features covered by hypermethylated DMRs while green bars for hypomethylated DMRs. (**B),** Relative enrichment of hypermethylated ultra DMRs in different genomic features, which was carried out on the number of ultra DMRs in each genomic feature compared to the expected number. Features with significant relative enrichment (FDR<0.01) and odds ratio larger than one are highlight in red stars. The x-axis shows the odds ratio; the y-axis, the relevant genomic features.

**Supplemental Figure S5. Ultra DMRs are associated with gene- and CGI-related regions in SZ. (A),** Bar chart representing the number of each genomic feature with methylation signals. Red bar shows the number of features covered by hypermethylated DMRs while green bars for hypomethylated DMRs. (**B),** Relative enrichment of hypermethylated ultra DMRs in different genomic features, which was carried out on the number of ultra DMRs in each genomic feature compared to the expected number. Features with significant relative enrichment (FDR<0.01) and odds ratio larger than one are highlight in red stars. The x-axis shows the odds ratio; the y-axis, the relevant genomic features.

**Supplemental Figure S6. Ultra DMRs are associated with long CGIs and HCPs.** (**A),** Relative enrichment of hypomethylated ultra DMRs. (**B),** Hyper- or hypo-methylated ultra DMRs are enriched in LCGI promoters. (**C),** The distribution of CpG ratio for differential methylated promoters. (**D),** Hypermethylated ultra DMRs are enriched in HCPs.

**Supplemental Figure S7. Common genes aberrant methylated in SZs and BPs. (A),**Venny plot shows the common genes aberrant methylated in SZ and BPs. **(B)**, The protein interaction network consisted of genes aberrant methylated in SZs and BPs, yellow circles represent BP-associated genes, green circles represent SZ-associated genes, red circles are intersect. **(C)**, The degree distribution of aberrant methylated genes. **(D)**, The betweenness distribution of aberrant methylated genes. **(E)**, The closeness distribution of aberrant methylated genes.

**Supplementary Table 1.The hypermethylated and hypomethylated promoters in SZs.**

**Supplementary Table 2.The Specific hypermethylated and hypomethylated promoters with distinct patterns in SZs.**

**Supplementary Table 3. The hypermethylated and hypomethylated promoters in BPs.**

**Supplementary Table 4. KEGG pathways enriched by aberrant methylated genes specifically in SZs/normal.**

**Supplementary Table 5. KEGG pathways enriched by aberrant methylated genes in BPs.**

**Supplementary Table 6. Aberrant hypermethylated HCP genes in SZs.**

**Supplementary text**

**Clinical Peripheral Blood samples**

Genomic DNA was extracted from blood created as detailed in a previous report. These data and samples were collected over the last ten years via three recent NIMH grants on the Genetics of Bipolar Disorder (BP) and Schizophrenia (SZ) in Latino populations [NIH-R01 grants, PI: Dr. Escamilla, Texas Tech University Health Science Center (TTUHSC)]. Subjects have been and will be continuously recruited through systematic screening of outpatient and inpatient facilities to increase statistical power to identify disease risk variants. All of the patients included as affecteds in this analysis have met best estimate consensus diagnoses of BPI or SZ by DSM-IV-TR criteria, as previously reported. Lifetime history of psychosis was defined as presence of criteria A of SZ as per the DSM-IV-TR. Age at onset of illness was defined as the first distinct episode meeting DSM-IV criteria for depression or hypomania⁄mania. The best estimators independently rated quantitative measures of psychosis and mood symptoms experienced by subjects as indicated in the Lifetime Dimension of Psychosis Scale. These studies have been approved by the Institutional Review Board of TTUHSC and participating institutions in the United States. Written informed consent was obtained from all the participants.

**MeDIP-Seq**

This experiment of MeDIP-Seq was conducted at one of the world biggest biotech company, BGI (<http://www.genomics.cn/en/index>) and the method was the same as a previously reported. Briefly, genome DNA was fragmented to 100-500bp by sonication. DNA-end was repaired to overhang a 3′-dA, and then adapters were ligated to the end of DNA fragments. Double-stranded DNA was denatured. Then DNA fragments were immunoprecipitated by 5mC antibody. Real-time PCR was used to validate the quality of immunoprecipitation. DNA fragments with proper size (usually 200-300bp, including adaptor sequence) were selected after PCR amplification. Finally we got qualified library for sequencing.

The human genome sequence and mapping information are downloaded from the University of California Santa Cruz Genome Bioinformatics Site (UCSC, <http://genome.ucsc.edu/>). MeDIP-Seq data was mapped to the reference genome using the software SOAP2, only unique alignments with no more than 2 mismatches were considered in further analysis.

**Gene expression analysis**

RNA-seq was performed to profile the gene expression in one normal and three schizophrenia samples. Clean reads were mapped to reference genome and genes sequences respectively using SOAP2, mismatches no more than 5 bases were allowed in the alignment. And then the reads per kilobase of model per million base pairs sequenced (RPKM) was used to quantify the gene expression level described in detail by Mortazavi et al. If genes are with more than one transcript, the longest one is used to calculate its expression level.

**Gene set enrichment analysis**

Gene Set Enrichment Analysis (GSEA) is a computational method that determines whether an a priori defined set of genes shows statistically significant, concordant differences between two biological states (e.g. phenotypes). The gene expression from normal and SZ profiled by RNA-seq was uploaded to GSEA, and enrichment analysis was performed using aberrant methylation target gene lists, such as aberrant methylated HCPs, ICP, LCPs or gene sets with distinct aberrant methylation patterns.

**Functional pathways identification**

KEGG analysis was performed to find enriched pathways using the Gene Set Analysis Toolkit V2 (<http://bioinfo.vanderbilt.edu/webgestalt/>), which is based on hypergeometric test. P-values were multiple tests corrected in order to reduce false-positive rates. KEGG pathways with p_adjusted_-values of <0.05 and with at least two interesting genes were considered significant.

**Protein-protein interaction network and topological features analyses**

The human protein-protein interaction (PPI) data were downloaded from Human Protein Reference Database (HPRD, release 9). After removing entries that lacked Entrez gene identifiers and only considering the maximum component of the interactome, the PPIN we analysed contained 35,865 interactions among 9028 proteins.

Topological centrality in the PPIN can be characterized by three widely-used measures: degree, betweenness centrality and closeness centrality. Degree is defined as the number of connections it has to other genes. Betweenness centrality, which represents how influential a gene is in communicating between node pairs, is defined by$bc_{i}=\sum_{j<k} \frac{g_{jk}(i)}{g_{jk}}$, where $g_{jk}(i)$ is the number of shortest path between node *j* and *k* that across gene *i* and $g_{jk}$ is the total number of shortest paths connecting nodes *j* and *k*. Closeness centrality is defined as the mean shortest path between a gene and all other genes reachable from it. The greater these measures of a node are, the more central it is.

**References**

**1.	Gonzalez S., Xu C., Ramirez M., J. Z., Armas R., Contreras S.A., Contreras-Rojas J., Dassori A., Leach R.J., Flores D., et al. 2011 Evidence for Association between L-Type Voltage-Gated Calcium Channel (CACNA1C) Gene Haplotypes and Bipolar Disorder in Latinos: a Family-Based Association Study**

**2.	Matsuo K., Walss-Bass C., Nery F.G., Nicoletti M.A., Hatch J.P., Frey B.N., Monkul E.S., Zunta-Soares G.B., Bowden C.L., Escamilla M.A., et al. 2009 Neuronal correlates of brain-derived neurotrophic factor Val66Met polymorphism and morphometric abnormalities in bipolar disorder. *Neuropsychopharmacology* 34(8), 1904-1913. (doi:10.1038/npp.2009.23).**

**3.	Glahn D.C., Almasy L., Barguil M., Hare E., Peralta J.M., Kent J.W., Jr., Dassori A., Contreras J., Pacheco A., Lanzagorta N., et al. 2010 Neurocognitive endophenotypes for bipolar disorder identified in multiplex multigenerational families. *Arch Gen Psychiatry* 67(2), 168-177. (doi:10.1001/archgenpsychiatry.2009.184).**

**4.	Jimenez-Castro L., Raventos-Vorst H., Escamilla M. 2011 Substance use disorder and schizophrenia: prevalence and sociodemographic characteristics in the Latin American population. *Actas espanolas de psiquiatria* 39(2), 123-130.**

**5.	Jimenez-Castro L., Hare E., Medina R., Raventos H., Nicolini H., Mendoza R., Ontiveros A., Jerez A., Munoz R., Dassori A., et al. 2010 Substance use disorder comorbidity with schizophrenia in families of Mexican and Central American ancestry. *Schizophr Res* 120(1-3), 87-94. (doi:10.1016/j.schres.2010.02.1053).**

**6.	Li N., Ye M., Li Y., Yan Z., Butcher L.M., Sun J., Han X., Chen Q., Zhang X., Wang J. 2010 Whole genome DNA methylation analysis based on high throughput sequencing technology. *Methods* 52(3), 203-212. (doi:10.1016/j.ymeth.2010.04.009).**

**7.	Mortazavi A., Schwarz E.M., Williams B., Schaeffer L., Antoshechkin I., Wold B.J., Sternberg P.W. 2010 Scaffolding a Caenorhabditis nematode genome with RNA-seq. *Genome Res* 20(12), 1740-1747. (doi:10.1101/gr.111021.110).**

**8.	Keshava Prasad T.S., Goel R., Kandasamy K., Keerthikumar S., Kumar S., Mathivanan S., Telikicherla D., Raju R., Shafreen B., Venugopal A., et al. 2009 Human Protein Reference Database--2009 update. *Nucleic acids research* 37(Database issue), D767-772. (doi:10.1093/nar/gkn892).**
