# Supplementary material for: Genome-Wide Methylome Analyses Reveal Novel Epigenetic Regulation Patterns in Schizophrenia and Bipolar Disorder
Source: Biomed Res Int. 2015 Feb 4;2015:201587. doi: 10.1155/2015/201587 (PMC4334857; doi:10.1155/2015/201587)
Supplement: Supplementary file 1 — Supplemental Figure S1: The DNA methylation landscape of bipolar disease. Supplemental Figure S2: Genomic distribution of DMRs in bipolar disease. Supplemental Figure S3: Ultra DMRs are associated with gene- and CGI-related regions. Supplemental Figure S4: Ultra DMRs are associated with gene- and CGI-related regions in BP. Supplemental Figure S5: Ultra DMRs are associated with gene- and CGI-related regions in SZ. Supplemental Figure S6: Ultra DMRs are associated with long CGIs and HCPs. Supplemental Figure S7: Common genes aberrant methylated in SZs and BPs. Supplementary Table 1:The hypermethylated and hypomethylated promoters in SZs. Supplementary Table 2:The Specific hypermethylated and hypomethylated promoters with distinct patterns in SZs. Supplementary Table 3: The hypermethylated and hypomethylated promoters in BPs. Supplementary Table 4: KEGG pathways enriched by aberrant methylated genes specifically in SZs/normal. Supplementary Table 5: KEGG pathways enriched by aberrant methylated genes in BPs. Supplementary Table 6: Aberrant hypermethylated HCP genes in SZs. [file 201587.f1.zip › Supplementary Table 2.doc]

**Supplementary Table 2. The Specific hypermethylated and hypomethylated promoters with distinct patterns in SZs.**

**Hypermethylated**

| **ID** | **Refseq** | **Chr** | **Start** | **End** | **Gene Symbol** | **Pattern** | **Reference** |
| --- | --- | --- | --- | --- | --- | --- | --- |
| 1 | NM_001145156 | 15 | 94676797 | 94684496 | NR2F2 | island |  |
| 2 | NM_178569 | 5 | 2805261 | 2808511 | C5orf38 | island |  |
| 3 | NM_001080461 | 7 | 1239179 | 1243139 | UNCX | island |  |
| 4 | NM_001145157 | 15 | 94677572 | 94684496 | NR2F2 | island |  |
| 5 | NM_001040429 | 13 | 57103789 | 57201066 | PCDH17 | island |  |
| 6 | NM_032829 | 12 | 108636569 | 108692695 | FAM222A | island |  |
| 7 | NM_001163678 | 3 | 159296493 | 159306646 | SHOX2 | island |  |
| 8 | NM_003030 | 3 | 159296493 | 159306646 | SHOX2 | island |  |
| 9 | NM_006884 | 3 | 159296493 | 159306646 | SHOX2 | island |  |
| 10 | NM_007197 | 12 | 129212956 | 129216238 | FZD10 | island |  |
| 11 | NM_001034914 | 2 | 95376494 | 95415552 | KCNIP3 | island |  |
| 12 | NM_001105541 | 8 | 24828359 | 24832511 | NEFM | island |  |
| 13 | NM_000524 | 5 | 63292033 | 63293302 | HTR1A | island |  |
| 14 | NM_021025 | 5 | 170668892 | 170671743 | TLX3 | island |  |
| 15 | NM_001260508 | 2 | 155263338 | 155423110 | KCNJ3 | island |  |
| 16 | NM_001260509 | 2 | 155263338 | 155274608 | KCNJ3 | island |  |
| 17 | NM_001260510 | 2 | 155263338 | 155264349 | KCNJ3 | island |  |
| 18 | NM_002239 | 2 | 155263338 | 155423110 | KCNJ3 | island |  |
| 19 | NM_030929 | 10 | 102810988 | 102815341 | KAZALD1 | island |  |
| 20 | NM_000684 | 10 | 115793795 | 115796657 | ADRB1 | island |  |
| 21 | NM_004852 | 18 | 53253914 | 53309528 | ONECUT2 | island |  |
| 22 | NM_002653 | 5 | 134391322 | 134397863 | PITX1 | island |  |
| 23 | NM_005382 | 8 | 24827178 | 24832511 | NEFM | island |  |
| 24 | NM_031424 | 20 | 773284 | 774922 | FAM110A | island |  |
| 25 | NM_001099336 | 12 | 102219862 | 102413879 | C12orf42 | island |  |
| 26 | NM_198521 | 12 | 102219862 | 102413876 | C12orf42 | island |  |
| 27 | NM_207645 | 11 | 108798055 | 108805103 | C11orf87 | island |  |
| 28 | NM_007113 | 1 | 150345416 | 150353180 | TCHH | island |  |
| 29 | NM_005322 | 6 | 27942548 | 27943338 | HIST1H1B | 5' shore |  |
| 30 | NM_001253380 | 3 | 23934199 | 23937351 | RPL15 | 5' shore |  |
| 31 | NM_001207068 | 2 | 201385300 | 201395211 | BZW1 | 5' shore |  |
| 32 | NM_001172504 | 16 | 54248056 | 54297605 | SLC6A2 | 5' shore |  |
| 33 | NM_001207069 | 2 | 201385152 | 201396814 | BZW1 | 5' shore |  |
| 34 | NM_033254 | 3 | 114414064 | 114488995 | BOC | 5' shore |  |
| 35 | NM_006808 | 9 | 101024390 | 101032722 | SEC61B | 5' shore |  |
| 36 | NM_001043 | 16 | 54247852 | 54295201 | SLC6A2 | 5' shore |  |
| 37 | NM_001099695 | 7 | 149696811 | 149702066 | REPIN1 | 5' shore |  |
| 38 | NM_001099696 | 7 | 149696811 | 149702066 | REPIN1 | 5' shore |  |
| 39 | NM_013400 | 7 | 149696811 | 149702066 | REPIN1 | 5' shore |  |
| 40 | NM_021809 | 20 | 34635423 | 34655769 | TGIF2 | 5' shore |  |
| 41 | NM_001253383 | 3 | 23933621 | 23937351 | RPL15 | 5' shore |  |
| 42 | NM_005921 | 5 | 56146656 | 56227735 | MAP3K1 | 5' shore |  |
| 43 | NM_001199513 | 20 | 34635289 | 34655769 | TGIF2 | 5' shore |  |
| 44 | NM_001199514 | 20 | 34635289 | 34655769 | TGIF2 | 5' shore |  |
| 45 | NM_032827 | 2 | 85834419 | 85872017 | ATOH8 | 5' shore |  |
| 46 | NM_004295 | 17 | 24095149 | 24102103 | TRAF4 | 5' shore |  |
| 47 | NM_001145191 | 4 | 15292449 | 15301168 | FAM200B | 5' shore |  |
| 48 | NM_001558 | 11 | 117362315 | 117377408 | IL10RA | 5' shore |  |
| 49 | NM_001145511 | 1 | 61315533 | 61701048 | NFIA | 5' shore |  |
| 50 | NM_001253379 | 3 | 23933298 | 23937351 | RPL15 | 5' shore |  |
| 51 | NM_001253384 | 3 | 23933298 | 23940191 | RPL15 | 5' shore |  |
| 52 | NM_002948 | 3 | 23933298 | 23937351 | RPL15 | 5' shore |  |
| 53 | NM_005435 | 7 | 143683421 | 143708658 | ARHGEF5 | 5' shore |  |
| 54 | NM_003407 | 19 | 44589326 | 44591885 | ZFP36 | 5' shore |  |
| 55 | NM_001165037 | 15 | 24663365 | 24777103 | GABRA5 | 5' shore |  |
| 56 | NM_001207067 | 2 | 201384513 | 201396814 | BZW1 | 5' shore |  |
| 57 | NM_014670 | 2 | 201384513 | 201396814 | BZW1 | 5' shore |  |
| 58 | NM_025092 | 11 | 279137 | 285688 | ATHL1 | 5' shore |  |
| 59 | NM_001130440 | 1 | 224032137 | 224044791 | SRP9 | 5' shore |  |
| 60 | NM_003133 | 1 | 224032137 | 224044791 | SRP9 | 5' shore |  |
| 61 | NM_021222 | 1 | 149247596 | 149274813 | PRUNE | 5' shore |  |
| 62 | NM_002166 | 2 | 8739563 | 8742034 | ID2 | 5' shore |  |
| 63 | NM_014394 | 10 | 85889164 | 85903291 | GHITM | 5' shore |  |
| 64 | NM_004823 | 19 | 43502323 | 43511489 | KCNK6 | 5' shore |  |
| 65 | NM_003545 | 6 | 26312851 | 26313228 | HIST1H4E | 5' shore |  |
| 66 | NM_001114393 | 5 | 78943998 | 79018227 | PAPD4 | 5' shore |  |
| 67 | NM_001114394 | 5 | 78943998 | 79018227 | PAPD4 | 5' shore |  |
| 68 | NM_173797 | 5 | 78943998 | 79018227 | PAPD4 | 5' shore |  |
| 69 | NM_001004065 | 9 | 111850698 | 111974612 | AKAP2 | 5' shore |  |
| 70 | NM_001198656 | 9 | 111850698 | 111974612 | AKAP2 | 5' shore |  |
| 71 | NM_001172501 | 16 | 54247042 | 54295201 | SLC6A2 | 5' shore |  |
| 72 | NM_152679 | 4 | 48180116 | 48186298 | SLC10A4 | 5' shore |  |
| 73 | NM_021630 | 8 | 22492198 | 22507755 | PDLIM2 | 5' shore |  |
| 74 | NM_000565 | 1 | 152644292 | 152708550 | IL6R | 5' shore |  |
| 75 | NM_001206866 | 1 | 152644292 | 152677185 | IL6R | 5' shore |  |
| 76 | NM_181359 | 1 | 152644292 | 152708550 | IL6R | 5' shore |  |
| 77 | NM_003511 | 6 | 27941085 | 27941555 | HIST1H2AL | 5' shore |  |
| 78 | NM_000810 | 15 | 24662958 | 24777103 | GABRA5 | 5' shore |  |
| 79 | NM_003636 | 1 | 6008659 | 6083840 | KCNAB2 | 5' shore |  |
| 80 | NM_172130 | 1 | 6008659 | 6083840 | KCNAB2 | 5' shore |  |
| 81 | NM_001100817 | 18 | 42802655 | 42804296 | TCEB3CL | 5' shore |  |
| 82 | NM_001242907 | 18 | 42802655 | 42804532 | LOC100506888 | 5' shore |  |
| 83 | NM_145653 | 18 | 42802655 | 42804532 | TCEB3C | 5' shore |  |
| 84 | NM_000211 | 21 | 45130296 | 45165393 | ITGB2 | 5' shore |  |
| 85 | NM_001127491 | 21 | 45130296 | 45173181 | ITGB2 | 5' shore |  |
| 86 | NM_012374 | 17 | 53587513 | 53588446 | OR4D1 | 5' shore |  |
| 87 | NM_173211 | 18 | 3443771 | 3448406 | TGIF1 | 3' shore |  |
| 88 | NM_033066 | 2 | 202217841 | 202271662 | MPP4 | 3' shore |  |
| 89 | NM_022140 | 5 | 111526213 | 111782909 | EPB41L4A | 3' shore |  |
| 90 | NM_170695 | 18 | 3441590 | 3448406 | TGIF1 | 3' shore |  |
| 91 | NM_173210 | 18 | 3441590 | 3448406 | TGIF1 | 3' shore |  |
| 92 | NM_021034 | 11 | 309672 | 310914 | IFITM3 | 3' shore |  |
| 93 | NM_016628 | 10 | 28862426 | 28952047 | WAC | 3' shore |  |
| 94 | NM_100486 | 10 | 28862426 | 28952047 | WAC | 3' shore |  |
| 95 | NM_001677 | 1 | 167342570 | 167368584 | ATP1B1 | 3' shore |  |
| 96 | NM_080606 | 20 | 61107775 | 61108832 | BHLHE23 | 3' shore |  |
| 97 | NM_007224 | 12 | 55896844 | 55906499 | NXPH4 | 3' shore |  |
| 98 | NM_173800 | 5 | 115326049 | 115391198 | AQPEP | 3' shore |  |
| 99 | NM_002224 | 6 | 33697133 | 33772326 | ITPR3 | 3' shore |  |
| 100 | NM_022045 | 8 | 121526846 | 121605056 | MTBP | 3' shore |  |
| 101 | NM_152386 | 2 | 222997565 | 223131861 | SGPP2 | 3' shore |  |
| 102 | NM_018928 | 5 | 140844924 | 140872730 | PCDHGC4 | 3' shore |  |
| 103 | NM_032406 | 5 | 140844924 | 140847540 | PCDHGC4 | 3' shore |  |
| 104 | NM_001130851 | 14 | 53933422 | 53956684 | CDKN3 | 3' shore |  |
| 105 | NM_005192 | 14 | 53933422 | 53956684 | CDKN3 | 3' shore |  |
| 106 | NM_001242644 | 8 | 61592022 | 61698757 | RAB2A | 3' shore |  |
| 107 | NM_002865 | 8 | 61592022 | 61698757 | RAB2A | 3' shore |  |
| 108 | NM_018248 | 4 | 178467984 | 178521086 | NEIL3 | 3' shore |  |
| 109 | NM_001145199 | 12 | 104248543 | 104289426 | C12orf75 | 3' shore |  |
| 110 | NM_003796 | 19 | 35124985 | 35199359 | URI1 | 3' shore |  |
| 111 | NM_002953 | 1 | 26728835 | 26774107 | RPS6KA1 | 3' shore |  |
| 112 | NM_153633 | 12 | 52733927 | 52736081 | HOXC4 | overlap |  |
| 113 | NM_022006 | 19 | 40325993 | 40337045 | FXYD7 | overlap |  |
| 114 | NM_080840 | 20 | 2802141 | 2967315 | PTPRA | overlap |  |
| 115 | NM_002517 | 19 | 52215982 | 52240857 | NPAS1 | overlap |  |
| 116 | NM_001145335 | 15 | 73923439 | 73980443 | UBE2Q2 | overlap |  |
| 117 | NM_020143 | 2 | 68238508 | 68256595 | PNO1 | overlap |  |
| 118 | NM_016463 | 5 | 139008484 | 139042864 | CXXC5 | overlap |  |
| 119 | NM_001253382 | 3 | 23933656 | 23937351 | RPL15 | overlap |  |
| 120 | NM_021116 | 7 | 45580649 | 45729239 | ADCY1 | overlap |  |
| 121 | NM_001143757 | 10 | 133995730 | 134045000 | LRRC27 | overlap |  |
| 122 | NM_004379 | 2 | 208102860 | 208178529 | CREB1 | overlap |  |
| 123 | NM_134442 | 2 | 208102860 | 208178529 | CREB1 | overlap |  |
| 124 | NM_001143758 | 10 | 133995603 | 134016891 | LRRC27 | overlap |  |
| 125 | NM_001143759 | 10 | 133995603 | 134016891 | LRRC27 | overlap |  |
| 126 | NM_030626 | 10 | 133995603 | 134045000 | LRRC27 | overlap |  |
| 127 | NM_001098503 | 11 | 47958685 | 48110842 | PTPRJ | overlap |  |
| 128 | NM_002843 | 11 | 47958685 | 48148970 | PTPRJ | overlap |  |
| 129 | NM_001135058 | 14 | 30413491 | 30429573 | COCH | overlap |  |
| 130 | NM_004086 | 14 | 30413491 | 30429573 | COCH | overlap |  |
| 131 | NM_173469 | 15 | 73922676 | 73980443 | UBE2Q2 | overlap |  |
| 132 | NM_173491 | 5 | 157103332 | 157116324 | LSM11 | overlap |  |
| 133 | NM_018683 | 20 | 47986320 | 48003829 | RNF114 | overlap |  |
| 134 | NM_173483 | 19 | 15480335 | 15524128 | CYP4F22 | overlap |  |
| 135 | NM_014170 | 3 | 114192489 | 114202911 | GTPBP8 | overlap |  |
| 136 | NM_138485 | 3 | 114192489 | 114202911 | GTPBP8 | overlap |  |
| 137 | NM_015696 | 1 | 52840630 | 52847311 | GPX7 | overlap |  |
| 138 | NM_032905 | 10 | 6170954 | 6199428 | RBM17 | overlap |  |
| 139 | NM_032940 | 16 | 56054051 | 56063422 | POLR2C | overlap |  |
| 140 | NM_001161546 | 5 | 138755533 | 138758784 | C5orf65 | overlap |  |
| 141 | NM_001080209 | 18 | 5880183 | 5882103 | TMEM200C | overlap |  |
| 142 | NM_145260 | 2 | 19414726 | 19421853 | OSR1 | overlap |  |
| 143 | NM_004900 | 22 | 37708350 | 37718729 | APOBEC3B | no island |  |
| 144 | NM_000462 | 15 | 23133488 | 23235268 | UBE3A | no island |  |
| 145 | NM_130838 | 15 | 23133488 | 23201746 | UBE3A | no island |  |
| 146 | NM_130839 | 15 | 23133488 | 23235268 | UBE3A | no island |  |
| 147 | NM_001123228 | 3 | 153540176 | 153541469 | TMEM14E | no island |  |
| 148 | NM_007275 | 3 | 50337344 | 50340672 | TUSC2 | no island |  |
| 149 | NM_001206847 | 8 | 30615658 | 30623011 | LOC100507341 | no island |  |
| 150 | NM_000623 | 14 | 95740887 | 95780419 | BDKRB2 | no island |  |
| 151 | NM_152313 | 11 | 92520497 | 92570743 | SLC36A4 | no island |  |
| 152 | NM_001023567 | 15 | 32604775 | 32615514 | GOLGA8B | no island |  |
| 153 | NM_005737 | 2 | 235066424 | 235070432 | ARL4C | no island |  |
| 154 | NM_206966 | 5 | 147252463 | 147266294 | C5orf46 | no island |  |
| 155 | NM_004686 | 8 | 17198676 | 17315411 | MTMR7 | no island |  |
| 156 | NM_024091 | 5 | 7912271 | 7922150 | FASTKD3 | no island |  |
| 157 | NM_138370 | 2 | 42128664 | 42139172 | PKDCC | no island |  |
| 158 | NM_001122979 | 1 | 205197934 | 205210593 | FCAMR | no island |  |
| 159 | NM_001170631 | 1 | 205197934 | 205210593 | FCAMR | no island |  |
| 160 | NM_032029 | 1 | 205197934 | 205210593 | FCAMR | no island |  |
| 161 | NM_001142678 | 12 | 43972732 | 44112401 | ANO6 | no island |  |
| 162 | NM_198152 | 3 | 192467637 | 192531019 | UTS2D | no island |  |
| 163 | NM_001254949 | 4 | 95393467 | 95431466 | SMARCAD1 | no island |  |
| 164 | NM_000089 | 7 | 93861808 | 93898480 | COL1A2 | no island |  |
| 165 | NM_153615 | 22 | 22363047 | 22371363 | RGL4 | no island |  |
| 166 | NM_001014291 | 1 | 151388681 | 151390051 | SPRR2G | no island |  |
| 167 | NM_001002925 | 11 | 56165540 | 56166491 | OR5AP2 | no island |  |
| 168 | NM_004673 | 1 | 177085292 | 177106838 | ANGPTL1 | no island |  |
| 169 | NM_013252 | 7 | 141273625 | 141293252 | CLEC5A | no island |  |
| 170 | NM_006330 | 8 | 55121490 | 55177130 | LYPLA1 | no island |  |
| 171 | NM_001040110 | 7 | 129057154 | 129184158 | NRF1 | no island |  |
| 172 | NM_000851 | 1 | 110056386 | 110062414 | GSTM5 | no island |  |
| 173 | NM_014981 | 3 | 109581905 | 109730859 | MYH15 | no island |  |
| 174 | NM_144984 | 10 | 49980222 | 49993565 | VSTM4 | no island |  |
| 175 | NM_182905 | 9 | 4510 | 19739 | WASH1 | no island |  |
| 176 | NM_001005188 | 11 | 123129497 | 123130436 | OR6X1 | no island |  |
| 177 | NM_172160 | 3 | 157321030 | 157739621 | KCNAB1 | no island |  |
| 178 | NM_001193502 | 8 | 68021289 | 68037379 | TCF24 | no island |  |
| 179 | NM_145301 | 17 | 15346302 | 15407670 | FAM18B2 | no island |  |
| 180 | NM_015842 | 13 | 75232797 | 75332007 | LMO7 | no island |  |
| 181 | NM_014207 | 11 | 60626505 | 60651899 | CD5 | no island |  |
| 182 | NM_024631 | 11 | 124141603 | 124175509 | MSANTD2 | no island |  |
| 183 | NM_015914 | 16 | 11680443 | 11744149 | TXNDC11 | no island |  |
| 184 | NM_014846 | 8 | 126105684 | 126173243 | KIAA0196 | no island |  |
| 185 | NM_006437 | 13 | 23893068 | 23984948 | PARP4 | no island |  |
| 186 | NM_031440 | 3 | 46514488 | 46517443 | RTP3 | no island |  |
| 187 | NM_015376 | 2 | 33592445 | 33643302 | RASGRP3 | no island |  |
| 188 | NM_020165 | 3 | 8893879 | 8980159 | RAD18 | no island |  |
| 189 | NM_002820 | 12 | 28006520 | 28016183 | PTHLH | no island |  |
| 190 | NM_198964 | 12 | 28006520 | 28014161 | PTHLH | no island |  |
| 191 | NM_138330 | 19 | 23627547 | 23661857 | ZNF675 | no island |  |
| 192 | NM_130772 | 5 | 76181581 | 76252812 | S100Z | no island |  |
| 193 | NM_004271 | 6 | 6533932 | 6600215 | LY86 | no island |  |
| 194 | NM_006683 | 14 | 20283938 | 20286379 | EDDM3A | no island |  |
| 195 | NM_018843 | 7 | 87301749 | 87343628 | SLC25A40 | no island |  |
| 196 | NM_001145645 | 13 | 107719977 | 107758833 | TNFSF13B | no island |  |
| 197 | NM_006573 | 13 | 107719977 | 107758833 | TNFSF13B | no island |  |
| 198 | NM_001242704 | 17 | 37431119 | 37443570 | ZNF385C | no island |  |
| 199 | NM_001168411 | 6 | 73133152 | 73169566 | RIMS1 | no island |  |
| 200 | NM_000040 | 11 | 116205833 | 116208997 | APOC3 | no island |  |
| 201 | NM_001039083 | 17 | 41772521 | 41794876 | ARL17B | no island |  |
| 202 | NM_001005198 | 11 | 123639932 | 123640973 | OR8G5 | no island |  |
| 203 | NM_001199880 | 8 | 22601118 | 22605847 | EGR3 | no island |  |
| 204 | NM_001199881 | 8 | 22601118 | 22606038 | EGR3 | no island |  |
| 205 | NM_004430 | 8 | 22601118 | 22606760 | EGR3 | no island |  |
| 206 | NM_001199377 | 15 | 76250241 | 76314104 | ACSBG1 | no island |  |
| 207 | NM_015162 | 15 | 76250241 | 76314104 | ACSBG1 | no island |  |
| 208 | NM_032810 | 10 | 89502854 | 89567897 | ATAD1 | no island |  |
| 209 | NM_001005566 | 11 | 57946313 | 57947362 | OR5B2 | no island |  |
| 210 | NM_002519 | 11 | 107533327 | 107598575 | NPAT | no island |  |
| 211 | NM_001040107 | 12 | 109570873 | 109612000 | HVCN1 | no island |  |
| 212 | NM_001256413 | 12 | 109570873 | 109612000 | HVCN1 | no island |  |
| 213 | NM_032369 | 12 | 109570873 | 109611345 | HVCN1 | no island |  |
| 214 | NM_053283 | 12 | 53324641 | 53328416 | DCD | no island |  |
| 215 | NM_152637 | 12 | 54361596 | 54364661 | METTL7B | no island |  |
| 216 | NM_000119 | 15 | 41276717 | 41300615 | EPB42 | no island |  |
| 217 | NM_001114134 | 15 | 41276717 | 41300615 | EPB42 | no island |  |
| 218 | NM_002201 | 15 | 86983042 | 86999883 | ISG20 | no island |  |
| 219 | NM_030632 | 18 | 29412538 | 29581397 | ASXL3 | no island |  |
| 220 | NM_002500 | 2 | 182249077 | 182253637 | NEUROD1 | no island |  |
| 221 | NM_001543 | 5 | 149867866 | 149917966 | NDST1 | no island |  |
| 222 | NM_018295 | 7 | 134483305 | 134501507 | TMEM140 | no island |  |
| 223 | NM_001925 | 8 | 6780754 | 6783196 | DEFA4 | no island |  |
| 224 | NM_001097635 | 9 | 78283076 | 78312152 | GCNT1 | no island |  |

Hypomethylated

| **ID** | **Refseq** | **Chr** | **Start** | **End** | **Gene Symbol** | **Pattern** | **Reference** |
| --- | --- | --- | --- | --- | --- | --- | --- |
| 1 | NM_001130183 | 15 | 76345614 | 76361593 | DNAJA4 | island |  |
| 2 | NM_017806 | 20 | 61838421 | 61840904 | LIME1 | island |  |
| 3 | NM_001099289 | 2 | 109112428 | 109619489 | SH3RF3 | island |  |
| 4 | NM_001130182 | 15 | 76344043 | 76361593 | DNAJA4 | island |  |
| 5 | NM_001010872 | 6 | 54819527 | 54914778 | FAM83B | island |  |
| 6 | NM_001025366 | 6 | 43845923 | 43862201 | VEGFA | island |  |
| 7 | NM_001025367 | 6 | 43845923 | 43862201 | VEGFA | island |  |
| 8 | NM_001025368 | 6 | 43845923 | 43862201 | VEGFA | island |  |
| 9 | NM_001025369 | 6 | 43845923 | 43862201 | VEGFA | island |  |
| 10 | NM_001025370 | 6 | 43845923 | 43862201 | VEGFA | island |  |
| 11 | NM_001033756 | 6 | 43845923 | 43862201 | VEGFA | island |  |
| 12 | NM_001171622 | 6 | 43845923 | 43862201 | VEGFA | island |  |
| 13 | NM_001171623 | 6 | 43845923 | 43862201 | VEGFA | island |  |
| 14 | NM_001171624 | 6 | 43845923 | 43862201 | VEGFA | island |  |
| 15 | NM_001171625 | 6 | 43845923 | 43862201 | VEGFA | island |  |
| 16 | NM_001171626 | 6 | 43845923 | 43862201 | VEGFA | island |  |
| 17 | NM_001171627 | 6 | 43845923 | 43862201 | VEGFA | island |  |
| 18 | NM_001171628 | 6 | 43845923 | 43862201 | VEGFA | island |  |
| 19 | NM_001171629 | 6 | 43845923 | 43862201 | VEGFA | island |  |
| 20 | NM_001171630 | 6 | 43845923 | 43862201 | VEGFA | island |  |
| 21 | NM_001204384 | 6 | 43845923 | 43862201 | VEGFA | island |  |
| 22 | NM_001204385 | 6 | 43845923 | 43862201 | VEGFA | island |  |
| 23 | NM_003376 | 6 | 43845923 | 43862201 | VEGFA | island |  |
| 24 | NM_001258390 | 11 | 110916442 | 110937680 | LAYN | island |  |
| 25 | NM_001258391 | 11 | 110916442 | 110937680 | LAYN | island |  |
| 26 | NM_178834 | 11 | 110916442 | 110937680 | LAYN | island |  |
| 27 | NM_005576 | 15 | 72005841 | 72031531 | LOXL1 | island |  |
| 28 | NM_018602 | 15 | 76343541 | 76361593 | DNAJA4 | island |  |
| 29 | NM_001080464 | 14 | 103621775 | 103648799 | ASPG | island |  |
| 30 | NM_004378 | 15 | 76419720 | 76427627 | CRABP1 | island |  |
| 31 | NM_001242680 | 19 | 22261091 | 22291818 | ZNF729 | island |  |
| 32 | NM_001100 | 1 | 227633615 | 227636466 | ACTA1 | island |  |
| 33 | NM_022049 | 1 | 100776315 | 100780171 | GPR88 | island |  |
| 34 | NM_032195 | 21 | 33837219 | 33854843 | SON | 5' shore |  |
| 35 | NM_138927 | 21 | 33837219 | 33871682 | SON | 5' shore |  |
| 36 | NM_001046 | 5 | 127447381 | 127553279 | SLC12A2 | 5' shore |  |
| 37 | NM_001256461 | 5 | 127447381 | 127553279 | SLC12A2 | 5' shore |  |
| 38 | NM_024808 | 13 | 72200042 | 72228329 | BORA | 5' shore |  |
| 39 | NM_001024226 | 1 | 226336983 | 226353536 | ARF1 | 5' shore |  |
| 40 | NM_001658 | 1 | 226336983 | 226353536 | ARF1 | 5' shore |  |
| 41 | NM_001137673 | 19 | 10625936 | 10657443 | ILF3 | 5' shore |  |
| 42 | NM_004516 | 19 | 10625936 | 10657443 | ILF3 | 5' shore |  |
| 43 | NM_012218 | 19 | 10625936 | 10664095 | ILF3 | 5' shore |  |
| 44 | NM_017620 | 19 | 10625936 | 10664095 | ILF3 | 5' shore |  |
| 45 | NM_153464 | 19 | 10625936 | 10657443 | ILF3 | 5' shore |  |
| 46 | NM_018031 | 3 | 49019640 | 49028390 | WDR6 | 5' shore |  |
| 47 | NM_001008409 | 20 | 29922165 | 29994519 | TTLL9 | 5' shore |  |
| 48 | NM_014952 | 15 | 38520702 | 38547733 | BAHD1 | 5' shore |  |
| 49 | NM_033510 | 15 | 38437725 | 38450548 | DISP2 | 5' shore |  |
| 50 | NM_006178 | 17 | 42023350 | 42189995 | NSF | 5' shore |  |
| 51 | NM_001197098 | 9 | 33740818 | 33789229 | PRSS3 | 5' shore |  |
| 52 | NM_001124758 | 17 | 4348877 | 4389977 | SPNS2 | 5' shore |  |
| 53 | NM_000690 | 12 | 110689073 | 110732172 | ALDH2 | 5' shore |  |
| 54 | NM_001204889 | 12 | 110689073 | 110732172 | ALDH2 | 5' shore |  |
| 55 | NM_052929 | 1 | 15446354 | 15597209 | FHAD1 | 5' shore |  |
| 56 | NM_001193646 | 19 | 55124211 | 55129005 | ATF5 | 5' shore |  |
| 57 | NM_001048194 | 1 | 28717331 | 28738295 | RCC1 | 5' shore |  |
| 58 | NM_001048195 | 1 | 28717331 | 28738295 | RCC1 | 5' shore |  |
| 59 | NM_001269 | 1 | 28717331 | 28738295 | RCC1 | 5' shore |  |
| 60 | NM_015341 | 2 | 96365210 | 96405001 | NCAPH | 5' shore |  |
| 61 | NM_001243531 | 15 | 82632245 | 82641989 | LOC100505679 | 5' shore |  |
| 62 | NM_174908 | 3 | 192529567 | 192599153 | CCDC50 | 5' shore |  |
| 63 | NM_178335 | 3 | 192529567 | 192599153 | CCDC50 | 5' shore |  |
| 64 | NM_001204103 | 6 | 32229753 | 32239436 | PPT2 | 5' shore |  |
| 65 | NM_005155 | 6 | 32229753 | 32239436 | PPT2 | 5' shore |  |
| 66 | NM_001197097 | 9 | 33740463 | 33789229 | PRSS3 | 5' shore |  |
| 67 | NM_007343 | 9 | 33740463 | 33789229 | PRSS3 | 5' shore |  |
| 68 | NM_004946 | 5 | 168996828 | 169442964 | DOCK2 | 5' shore |  |
| 69 | NM_080672 | 20 | 57612997 | 57856161 | PHACTR3 | 5' shore |  |
| 70 | NM_138717 | 6 | 32229206 | 32239436 | PPT2 | 5' shore |  |
| 71 | NM_001199214 | 8 | 80685603 | 80740965 | STMN2 | 5' shore |  |
| 72 | NM_007029 | 8 | 80685603 | 80740965 | STMN2 | 5' shore |  |
| 73 | NM_001185022 | 17 | 7103945 | 7107236 | CLDN7 | 5' shore |  |
| 74 | NM_001185023 | 17 | 7103945 | 7106988 | CLDN7 | 5' shore |  |
| 75 | NM_001307 | 17 | 7103945 | 7106988 | CLDN7 | 5' shore |  |
| 76 | NM_002975 | 19 | 55918416 | 55920793 | CLEC11A | 5' shore |  |
| 77 | NM_004791 | 13 | 100902966 | 101166795 | ITGBL1 | 5' shore |  |
| 78 | NM_001160407 | 17 | 24075492 | 24078076 | TLCD1 | 5' shore |  |
| 79 | NM_138463 | 17 | 24075492 | 24077357 | TLCD1 | 5' shore |  |
| 80 | NM_001018070 | 11 | 66962093 | 66967868 | CORO1B | 3' shore |  |
| 81 | NM_020441 | 11 | 66962093 | 66967559 | CORO1B | 3' shore |  |
| 82 | NM_012109 | 19 | 18584681 | 18592849 | TMEM59L | 3' shore |  |
| 83 | NM_003619 | 4 | 119420640 | 119493370 | PRSS12 | 3' shore |  |
| 84 | NM_001123329 | 14 | 64041044 | 64061422 | ZBTB1 | 3' shore |  |
| 85 | NM_014950 | 14 | 64041044 | 64070161 | ZBTB1 | 3' shore |  |
| 86 | NM_002136 | 12 | 52960754 | 52965297 | HNRNPA1 | 3' shore |  |
| 87 | NM_031157 | 12 | 52960754 | 52965297 | HNRNPA1 | 3' shore |  |
| 88 | NM_016218 | 5 | 74843412 | 74931402 | POLK | 3' shore |  |
| 89 | NM_006086 | 16 | 88517187 | 88530006 | TUBB3 | 3' shore |  |
| 90 | NM_001135608 | 5 | 142130475 | 142588765 | ARHGAP26 | 3' shore |  |
| 91 | NM_015071 | 5 | 142130475 | 142588765 | ARHGAP26 | 3' shore |  |
| 92 | NM_001024844 | 11 | 44543716 | 44597891 | CD82 | 3' shore |  |
| 93 | NM_002231 | 11 | 44543716 | 44597891 | CD82 | 3' shore |  |
| 94 | NM_019058 | 10 | 73703682 | 73705803 | DDIT4 | 3' shore |  |
| 95 | NM_015352 | 20 | 30259356 | 30290128 | POFUT1 | 3' shore |  |
| 96 | NM_172236 | 20 | 30259356 | 30269387 | POFUT1 | 3' shore |  |
| 97 | NM_001164603 | 20 | 30409807 | 30424013 | ASXL1 | 3' shore |  |
| 98 | NM_015338 | 20 | 30409807 | 30490783 | ASXL1 | 3' shore |  |
| 99 | NM_015169 | 8 | 67503816 | 67505522 | RRS1 | 3' shore |  |
| 100 | NM_032288 | 3 | 198961020 | 198995714 | FYTTD1 | 3' shore |  |
| 101 | NM_001194954 | 5 | 138637339 | 138695265 | MATR3 | 3' shore |  |
| 102 | NM_199189 | 5 | 138637339 | 138695265 | MATR3 | 3' shore |  |
| 103 | NM_194318 | 13 | 30672111 | 30804411 | B3GALTL | 3' shore |  |
| 104 | NM_001011537 | 3 | 198960820 | 198995714 | FYTTD1 | 3' shore |  |
| 105 | NM_153340 | 1 | 109828083 | 109836943 | ATXN7L2 | 3' shore |  |
| 106 | NM_015656 | 14 | 103674812 | 103716988 | KIF26A | overlap |  |
| 107 | NM_015995 | 15 | 29406374 | 29457394 | KLF13 | overlap |  |
| 108 | NM_002763 | 1 | 212228482 | 212276385 | PROX1 | overlap |  |
| 109 | NM_001005915 | 12 | 54760158 | 54765668 | ERBB3 | overlap |  |
| 110 | NM_001982 | 12 | 54760075 | 54783558 | ERBB3 | overlap |  |
| 111 | NM_001080407 | 11 | 133651484 | 133694668 | GLB1L3 | overlap |  |
| 112 | NM_001003395 | 6 | 125517118 | 125626343 | TPD52L1 | overlap |  |
| 113 | NM_000142 | 4 | 1764836 | 1780397 | FGFR3 | overlap |  |
| 114 | NM_001163213 | 4 | 1764836 | 1780397 | FGFR3 | overlap |  |
| 115 | NM_022965 | 4 | 1764836 | 1780397 | FGFR3 | overlap |  |
| 116 | NM_001127207 | 2 | 216985717 | 217056019 | SMARCAL1 | overlap |  |
| 117 | NM_003442 | 11 | 9439087 | 9506647 | ZNF143 | overlap |  |
| 118 | NM_014141 | 7 | 145444385 | 147749021 | CNTNAP2 | overlap |  |
| 119 | NM_001184740 | 11 | 69922259 | 69960338 | CTTN | overlap |  |
| 120 | NM_005231 | 11 | 69922259 | 69960338 | CTTN | overlap |  |
| 121 | NM_138565 | 11 | 69922259 | 69960338 | CTTN | overlap |  |
| 122 | NM_001123066 | 17 | 41327541 | 41461546 | MAPT | overlap |  |
| 123 | NM_001123067 | 17 | 41327541 | 41461546 | MAPT | overlap |  |
| 124 | NM_001203251 | 17 | 41327541 | 41461546 | MAPT | overlap |  |
| 125 | NM_001203252 | 17 | 41327541 | 41461546 | MAPT | overlap |  |
| 126 | NM_005910 | 17 | 41327541 | 41461546 | MAPT | overlap |  |
| 127 | NM_016834 | 17 | 41327541 | 41461546 | MAPT | overlap |  |
| 128 | NM_016835 | 17 | 41327541 | 41461546 | MAPT | overlap |  |
| 129 | NM_016841 | 17 | 41327541 | 41461546 | MAPT | overlap |  |
| 130 | NM_001211 | 15 | 38240501 | 38300629 | BUB1B | overlap |  |
| 131 | NM_018352 | 4 | 164635122 | 164661141 | TMA16 | overlap |  |
| 132 | NM_015055 | 11 | 9642203 | 9731083 | SWAP70 | overlap |  |
| 133 | NM_000146 | 19 | 54160377 | 54161948 | FTL | overlap |  |
| 134 | NM_014140 | 2 | 216985381 | 217056019 | SMARCAL1 | overlap |  |
| 135 | NM_031286 | 1 | 26478799 | 26480600 | SH3BGRL3 | overlap |  |
| 136 | NM_002946 | 1 | 28090635 | 28113823 | RPA2 | no island |  |
| 137 | NM_020672 | 1 | 151853355 | 151855432 | S100A14 | no island |  |
| 138 | NM_001102653 | 4 | 146274251 | 146320282 | OTUD4 | no island |  |
| 139 | NM_207373 | 10 | 85923533 | 85935030 | C10orf99 | no island |  |
| 140 | NM_014991 | 4 | 85809716 | 86106568 | WDFY3 | no island |  |
| 141 | NM_181719 | 1 | 19881292 | 19998997 | TMCO4 | no island |  |
| 142 | NM_001630 | 10 | 47216925 | 47233046 | ANXA8L2 | no island |  |
| 143 | NM_152315 | 11 | 113897646 | 113935790 | FAM55A | no island |  |
| 144 | NM_000781 | 15 | 72417155 | 72447134 | CYP11A1 | no island |  |
| 145 | NM_001099773 | 15 | 72417155 | 72445606 | CYP11A1 | no island |  |
| 146 | NM_001173531 | 6 | 31240092 | 31242594 | POU5F1 | no island |  |
| 147 | NM_002701 | 6 | 31240092 | 31246430 | POU5F1 | no island |  |
| 148 | NM_203289 | 6 | 31240092 | 31242594 | POU5F1 | no island |  |
| 149 | NM_001074 | 4 | 69996781 | 70013294 | UGT2B7 | no island |  |
| 150 | NM_001004693 | 1 | 246822753 | 246823692 | OR2T10 | no island |  |
| 151 | NM_001145770 | 16 | 56211410 | 56256445 | GPR56 | no island |  |
| 152 | NM_005682 | 16 | 56211410 | 56256445 | GPR56 | no island |  |
| 153 | NM_015383 | 1 | 146470265 | 146492472 | NBPF14 | no island |  |
| 154 | NM_206861 | 10 | 123738678 | 124004047 | TACC2 | no island |  |
| 155 | NM_206862 | 10 | 123738678 | 124004047 | TACC2 | no island |  |
| 156 | NM_000035 | 9 | 103222662 | 103237883 | ALDOB | no island |  |
| 157 | NM_001167576 | 5 | 135576897 | 135729063 | TRPC7 | no island |  |
| 158 | NM_001167577 | 5 | 135576897 | 135729063 | TRPC7 | no island |  |
| 159 | NM_020389 | 5 | 135576897 | 135729063 | TRPC7 | no island |  |
| 160 | NM_004582 | 1 | 76024473 | 76033352 | RABGGTB | no island |  |
| 161 | NM_001098670 | 11 | 64250958 | 64268905 | RASGRP2 | no island |  |
| 162 | NM_001098671 | 11 | 64250958 | 64268206 | RASGRP2 | no island |  |
| 163 | NM_153819 | 11 | 64250958 | 64269504 | RASGRP2 | no island |  |
| 164 | NM_001177660 | 10 | 115300579 | 115339350 | HABP2 | no island |  |
| 165 | NM_032489 | 12 | 6617502 | 6626841 | ACRBP | no island |  |
| 166 | NM_006399 | 14 | 75058536 | 75083087 | BATF | no island |  |
| 167 | NM_001144966 | 18 | 54039733 | 54219752 | NEDD4L | no island |  |
| 168 | NM_004975 | 20 | 47421911 | 47532588 | KCNB1 | no island |  |
| 169 | NM_001040448 | 4 | 9055357 | 9061338 | DEFB131 | no island |  |
| 170 | NM_001030013 | 10 | 129237602 | 129240925 | NPS | no island |  |
| 171 | NM_000342 | 17 | 39681283 | 39701028 | SLC4A1 | no island |  |
| 172 | NM_015201 | 8 | 145456863 | 145485928 | BOP1 | no island |  |
| 173 | NM_001135240 | 1 | 160615319 | 160620125 | C1orf226 | no island |  |
| 174 | NM_012360 | 16 | 3194247 | 3195186 | OR1F1 | no island |  |
| 175 | NM_002087 | 17 | 39778016 | 39785996 | GRN | no island |  |
| 176 | NM_006032 | 14 | 23610595 | 23617136 | CPNE6 | no island |  |
| 177 | NM_001244638 | 10 | 63478975 | 63526713 | ARID5B | no island |  |
| 178 | NM_001190995 | 12 | 30673181 | 30721445 | IPO8 | no island |  |
| 179 | NM_006390 | 12 | 30673181 | 30740196 | IPO8 | no island |  |
| 180 | NM_174886 | 18 | 3402071 | 3448406 | TGIF1 | no island |  |
| 181 | NM_001164468 | 5 | 114942237 | 114989775 | TMED7-TICAM2 | no island |  |
| 182 | NM_001164469 | 5 | 114942237 | 114989775 | TMED7-TICAM2 | no island |  |
| 183 | NM_021649 | 5 | 114942237 | 114966075 | TICAM2 | no island |  |
| 184 | NM_001080538 | 7 | 133884388 | 133915132 | AKR1B15 | no island |  |
| 185 | NM_013289 | 19 | 60019704 | 60034045 | KIR3DL1 | no island |  |
| 186 | NM_001005200 | 11 | 55629094 | 55630033 | OR8H2 | no island |  |
| 187 | NM_001018082 | 20 | 36643251 | 36650518 | ADIG | no island |  |
| 188 | NM_001144952 | 17 | 68842117 | 69151822 | SDK2 | no island |  |
| 189 | NM_001193301 | 1 | 154386358 | 154414166 | SEMA4A | no island |  |
| 190 | NM_178431 | 1 | 150861933 | 150862203 | LCE3A | no island |  |
| 191 | NM_181604 | 21 | 30892875 | 30893064 | KRTAP6-2 | no island |  |
| 192 | NM_002190 | 6 | 52159143 | 52163395 | IL17A | no island |  |
| 193 | NM_006672 | 6 | 43373975 | 43381254 | SLC22A7 | no island |  |
| 194 | NM_153320 | 6 | 43373975 | 43381254 | SLC22A7 | no island |  |
| 195 | NM_001005194 | 11 | 123945174 | 123946155 | OR8A1 | no island |  |
| 196 | NM_001079935 | 19 | 9222719 | 9223739 | OR7E24 | no island |  |
| 197 | NM_031898 | 17 | 15147853 | 15185683 | TEKT3 | no island |  |
| 198 | NM_006080 | 7 | 83425594 | 83662153 | SEMA3A | no island |  |
| 199 | NM_001005241 | 15 | 19883836 | 19885179 | OR4N4 | no island |  |
| 200 | NM_001004757 | 11 | 5399916 | 5401012 | OR51Q1 | no island |  |
| 201 | NM_181684 | 21 | 44910533 | 44911272 | KRTAP12-2 | no island |  |
| 202 | NM_001079877 | 7 | 102007167 | 102044425 | RASA4 | no island |  |
| 203 | NM_006989 | 7 | 102007167 | 102044425 | RASA4 | no island |  |
| 204 | NM_001033667 | 1 | 159032487 | 159039160 | LY9 | no island |  |
| 205 | NM_001261456 | 1 | 159032487 | 159064669 | LY9 | no island |  |
| 206 | NM_001261457 | 1 | 159032487 | 159064669 | LY9 | no island |  |
| 207 | NM_002348 | 1 | 159032487 | 159064669 | LY9 | no island |  |
| 208 | NM_001032 | 14 | 49120039 | 49122844 | RPS29 | no island |  |
| 209 | NM_001142537 | 11 | 63032034 | 63040822 | LGALS12 | no island |  |
| 210 | NM_001142538 | 11 | 63032034 | 63040822 | LGALS12 | no island |  |
| 211 | NM_002964 | 1 | 151629131 | 151630288 | S100A8 | no island |  |
| 212 | NM_130446 | 3 | 184688012 | 184756193 | KLHL6 | no island |  |
| 213 | NM_001001824 | 1 | 246879854 | 246880808 | OR2T27 | no island |  |
| 214 | NM_152616 | 3 | 141879555 | 141902682 | TRIM42 | no island |  |
| 215 | NM_002725 | 1 | 201711505 | 201727102 | PRELP | no island |  |
| 216 | NM_201348 | 1 | 201711505 | 201727102 | PRELP | no island |  |
| 217 | NM_032184 | 1 | 85395943 | 85439316 | SYDE2 | no island |  |
| 218 | NM_014070 | 6 | 31186978 | 31188311 | C6orf15 | no island |  |
| 219 | NM_080877 | 9 | 139245205 | 139250827 | SLC34A3 | no island |  |
| 220 | NM_178826 | 12 | 99712504 | 100046550 | ANO4 | no island |  |
| 221 | NM_001177316 | 9 | 139245029 | 139250827 | SLC34A3 | no island |  |
| 222 | NM_001177317 | 9 | 139245029 | 139250827 | SLC34A3 | no island |  |
| 223 | NM_001633 | 9 | 115862228 | 115880573 | AMBP | no island |  |
| 224 | NM_018490 | 11 | 27344083 | 27450910 | LGR4 | no island |  |
| 225 | NM_002855 | 11 | 119036912 | 119104645 | PVRL1 | no island |  |
| 226 | NM_001145727 | 12 | 25540518 | 25692763 | IFLTD1 | no island |  |
| 227 | NM_138371 | 12 | 45896318 | 45916710 | FAM113B | no island |  |
| 228 | NM_001243328 | 6 | 150246203 | 150258888 | RAET1E | no island |  |
| 229 | NM_015258 | 9 | 114967620 | 115023462 | FKBP15 | no island |  |
| 230 | NM_002113 | 1 | 195055483 | 195067942 | CFHR1 | no island |  |
| 231 | NM_176820 | 19 | 60911609 | 60941580 | NLRP9 | no island |  |
| 232 | NM_001039083 | 17 | 41989935 | 42012375 | ARL17B | no island |  |
| 233 | NM_001870 | 3 | 150065732 | 150097562 | CPA3 | no island |  |
| 234 | NM_001153446 | 4 | 141484046 | 141523160 | SCOC | no island |  |
| 235 | NM_001153585 | 4 | 141484064 | 141523160 | SCOC | no island |  |
| 236 | NM_001153635 | 4 | 141484046 | 141523160 | SCOC | no island |  |
| 237 | NM_001153690 | 4 | 141484064 | 141523160 | SCOC | no island |  |
| 238 | NM_001079810 | 2 | 167453242 | 167824507 | XIRP2 | no island |  |
| 239 | NM_001199143 | 2 | 167453242 | 167824507 | XIRP2 | no island |  |
| 240 | NM_152381 | 2 | 167453242 | 167824507 | XIRP2 | no island |  |
| 241 | NM_144661 | 10 | 118413196 | 118419471 | C10orf82 | no island |  |
| 242 | NM_000764 | 19 | 46073183 | 46080497 | CYP2A7 | no island |  |
| 243 | NM_030589 | 19 | 46073183 | 46080497 | CYP2A7 | no island |  |
| 244 | NM_001285 | 1 | 86707113 | 86738562 | CLCA1 | no island |  |
| 245 | NM_173464 | 18 | 5944704 | 6404910 | L3MBTL4 | no island |  |
| 246 | NM_001160154 | 2 | 98602000 | 98646368 | MGAT4A | no island |  |
| 247 | NM_012214 | 2 | 98602000 | 98714021 | MGAT4A | no island |  |
| 248 | NM_001007253 | 7 | 64088167 | 64104559 | ERV3-1 | no island |  |
| 249 | NM_194313 | 9 | 34242377 | 34319198 | KIF24 | no island |  |
| 250 | NM_021798 | 16 | 27346079 | 27370864 | IL21R | no island |  |
| 251 | NM_207662 | 14 | 88130482 | 88149606 | ZC3H14 | no island |  |
| 252 | NM_006566 | 18 | 65681172 | 65775212 | CD226 | no island |  |

**References**

1.	Wockner, L.F., et al., *Genome-wide DNA methylation analysis of human brain tissue from schizophrenia patients.* Transl Psychiatry, 2014. **4**: p. e339.

2.	Dean, B., et al., *Gene expression profiling in Brodmann's area 46 from subjects with schizophrenia.* Aust N Z J Psychiatry, 2007. **41**(4): p. 308-20.

3.	Kekesi, K.A., et al., *Altered functional protein networks in the prefrontal cortex and amygdala of victims of suicide.* PLoS One, 2012. **7**(12): p. e50532.

4.	Zhou, X., et al., *Relationship between genetic polymorphisms in the HTR1A gene and paranoid schizophrenia in a northern Han Chinese population.* J Mol Neurosci, 2013. **49**(3): p. 625-31.

5.	Yamada, K., et al., *Association study of the KCNJ3 gene as a susceptibility candidate for schizophrenia in the Chinese population.* Hum Genet, 2012. **131**(3): p. 443-51.

6.	Kleinau, G., et al., *Differential modulation of Beta-adrenergic receptor signaling by trace amine-associated receptor 1 agonists.* PLoS One, 2011. **6**(10): p. e27073.

7.	Philippi, A., et al., *Association of autism with polymorphisms in the paired-like homeodomain transcription factor 1 (PITX1) on chromosome 5q31: a candidate gene analysis.* BMC Med Genet, 2007. **8**: p. 74.

8.	Aberg, K.A., et al., *Methylome-wide association study of schizophrenia: identifying blood biomarker signatures of environmental insults.* JAMA Psychiatry, 2014. **71**(3): p. 255-64.

9.	Yu, H., et al., *Protein-interaction-network-based analysis for genome-wide association analysis of schizophrenia in Han Chinese population.* J Psychiatr Res, 2014. **50**: p. 73-8.

10.	Meary, A., et al., *Pharmacogenetic study of atypical antipsychotic drug response: involvement of the norepinephrine transporter gene.* Am J Med Genet B Neuropsychiatr Genet, 2008. **147B**(4): p. 491-4.

11.	Shuvarikov, A., et al., *Recurrent HERV-H-mediated 3q13.2-q13.31 deletions cause a syndrome of hypotonia and motor, language, and cognitive delays.* Hum Mutat, 2013. **34**(10): p. 1415-23.

12.	Zeidan-Chulia, F., et al., *Altered expression of Alzheimer's disease-related genes in the cerebellum of autistic patients: a model for disrupted brain connectome and therapy.* Cell Death Dis, 2014. **5**: p. e1250.

13.	Aston, C., L. Jiang, and B.P. Sokolov, *Microarray analysis of postmortem temporal cortex from patients with schizophrenia.* J Neurosci Res, 2004. **77**(6): p. 858-66.

14.	Carter, C.J., *Schizophrenia susceptibility genes directly implicated in the life cycles of pathogens: cytomegalovirus, influenza, herpes simplex, rubella, and Toxoplasma gondii.* Schizophr Bull, 2009. **35**(6): p. 1163-82.

15.	Tsang, K.M., et al., *A genome-wide survey of transgenerational genetic effects in autism.* PLoS One, 2013. **8**(10): p. e76978.

16.	Thalmeier, A., et al., *Gene expression profiling of post-mortem orbitofrontal cortex in violent suicide victims.* Int J Neuropsychopharmacol, 2008. **11**(2): p. 217-28.

17.	Ribases, M., et al., *Case-control study of six genes asymmetrically expressed in the two cerebral hemispheres: association of BAIAP2 with attention-deficit/hyperactivity disorder.* Biol Psychiatry, 2009. **66**(10): p. 926-34.

18.	Borges, K., *Slc10A4 - what do we know about the function of this "secret ligand carrier" protein?* Exp Neurol, 2013. **248**: p. 258-61.

19.	Ban, J.Y., et al., *Association between Interleukin 31 Receptor A Gene Polymorphism and Schizophrenia in Korean Population.* Korean J Physiol Pharmacol, 2008. **12**(4): p. 205-9.

20.	Nardone, S., et al., *DNA methylation analysis of the autistic brain reveals multiple dysregulated biological pathways.* Transl Psychiatry, 2014. **4**: p. e433.

21.	Hwang, Y., et al., *Gene expression profiling by mRNA sequencing reveals increased expression of immune/inflammation-related genes in the hippocampus of individuals with schizophrenia.* Transl Psychiatry, 2013. **3**: p. e321.

22.	Kim, S. and M.J. Webster, *Integrative genome-wide association analysis of cytoarchitectural abnormalities in the prefrontal cortex of psychiatric disorders.* Mol Psychiatry, 2011. **16**(4): p. 452-61.

23.	Takahashi, N., et al., *Loss of function studies in mice and genetic association link receptor protein tyrosine phosphatase alpha to schizophrenia.* Biol Psychiatry, 2011. **70**(7): p. 626-35.

24.	Pieper, A.A., et al., *The neuronal PAS domain protein 3 transcription factor controls FGF-mediated adult hippocampal neurogenesis in mice.* Proc Natl Acad Sci U S A, 2005. **102**(39): p. 14052-7.

25.	Xiao, Y., et al., *The DNA methylome and transcriptome of different brain regions in schizophrenia and bipolar disorder.* PLoS One, 2014. **9**(4): p. e95875.

26.	Ma, L., et al., *Molecular evolution in the CREB1 signal pathway and a rare haplotype in CREB1 with genetic predisposition to schizophrenia.* J Psychiatr Res, 2014. **57**: p. 84-9.

27.	Yao, Y., J. Schroder, and H. Karlsson, *Verification of proposed peripheral biomarkers in mononuclear cells of individuals with schizophrenia.* J Psychiatr Res, 2008. **42**(8): p. 639-43.

28.	Forrest, M.P., et al., *Knockdown of human TCF4 affects multiple signaling pathways involved in cell survival, epithelial to mesenchymal transition and neuronal differentiation.* PLoS One, 2013. **8**(8): p. e73169.

29.	Otowa, T., et al., *Meta-analysis of genome-wide association studies for panic disorder in the Japanese population.* Transl Psychiatry, 2012. **2**: p. e186.

30.	Ishiura, H., et al., *Molecular epidemiology and clinical spectrum of hereditary spastic paraplegia in the Japanese population based on comprehensive mutational analyses.* J Hum Genet, 2014. **59**(3): p. 163-72.

31.	Manzardo, A.M., et al., *Exon microarray analysis of human dorsolateral prefrontal cortex in alcoholism.* Alcohol Clin Exp Res, 2014. **38**(6): p. 1594-601.

32.	Fulzele, S. and A. Pillai, *Decreased VEGF mRNA expression in the dorsolateral prefrontal cortex of schizophrenia subjects.* Schizophr Res, 2009. **115**(2-3): p. 372-3.

33.	Del Zompo, M., et al., *Association study in three different populations between the GPR88 gene and major psychoses.* Mol Genet Genomic Med, 2014. **2**(2): p. 152-9.

34.	Callicott, J.H., et al., *DISC1 and SLC12A2 interaction affects human hippocampal function and connectivity.* J Clin Invest, 2013. **123**(7): p. 2961-4.

35.	Hammond, J.C., et al., *Evidence for abnormal forward trafficking of AMPA receptors in frontal cortex of elderly patients with schizophrenia.* Neuropsychopharmacology, 2010. **35**(10): p. 2110-9.

36.	Zhang, F., et al., *A two-stage association study suggests BRAP as a susceptibility gene for schizophrenia.* PLoS One, 2014. **9**(1): p. e86037.

37.	Morris, J.A., et al., *DISC1 (Disrupted-In-Schizophrenia 1) is a centrosome-associated protein that interacts with MAP1A, MIPT3, ATF4/5 and NUDEL: regulation and loss of interaction with mutation.* Hum Mol Genet, 2003. **12**(13): p. 1591-608.

38.	Dahm, L., et al., *Seroprevalence of autoantibodies against brain antigens in health and disease.* Ann Neurol, 2014. **76**(1): p. 82-94.

39.	Stephens, S.H., et al., *Multiple genes in the 15q13-q14 chromosomal region are associated with schizophrenia.* Psychiatr Genet, 2012. **22**(1): p. 1-14.

40.	Yu, D.X., et al., *Modeling hippocampal neurogenesis using human pluripotent stem cells.* Stem Cell Reports, 2014. **2**(3): p. 295-310.

41.	Seshadri, S., et al., *Disrupted-in-Schizophrenia-1 expression is regulated by beta-site amyloid precursor protein cleaving enzyme-1-neuregulin cascade.* Proc Natl Acad Sci U S A, 2010. **107**(12): p. 5622-7.

42.	Karayannis, T., et al., *Cntnap4 differentially contributes to GABAergic and dopaminergic synaptic transmission.* Nature, 2014. **511**(7508): p. 236-40.

43.	Ji, W., et al., *CNTNAP2 is significantly associated with schizophrenia and major depression in the Han Chinese population.* Psychiatry Res, 2013. **207**(3): p. 225-8.

44.	Alkelai, A., et al., *Association of the type 2 diabetes mellitus susceptibility gene, TCF7L2, with schizophrenia in an Arab-Israeli family sample.* PLoS One, 2012. **7**(1): p. e29228.

45.	Galimberti, D., et al., *Progranulin gene variability and plasma levels in bipolar disorder and schizophrenia.* PLoS One, 2012. **7**(4): p. e32164.

46.	Chavarria-Siles, I., et al., *TGFB-induced factor (TGIF): a candidate gene for psychosis on chromosome 18p.* Mol Psychiatry, 2007. **12**(11): p. 1033-41.

47.	Fatemi, S.H., et al., *Maternal infection leads to abnormal gene regulation and brain atrophy in mouse offspring: implications for genesis of neurodevelopmental disorders.* Schizophr Res, 2008. **99**(1-3): p. 56-70.

48.	Xu, J., et al., *RNA-Seq analysis implicates dysregulation of the immune system in schizophrenia.* BMC Genomics, 2012. **13 Suppl 8**: p. S2.
